# Genetic Barriers to Historical Gene Flow between Cryptic Species of Alpine Bumblebees Revealed by Comparative Population Genomics

**DOI:** 10.1093/molbev/msab086

**Published:** 2021-04-06

**Authors:** Matthew J Christmas, Julia C Jones, Anna Olsson, Ola Wallerman, Ignas Bunikis, Marcin Kierczak, Valentina Peona, Kaitlyn M Whitley, Tuuli Larva, Alexander Suh, Nicole E Miller-Struttmann, Jennifer C Geib, Matthew T Webster

**Affiliations:** 1 Department of Medical Biochemistry and Microbiology, Science for Life Laboratory, Uppsala University, Uppsala, Sweden; 2 School of Biology and Environmental Science, University College Dublin, Dublin, Ireland; 3 Department of Immunology, Genetics and Pathology, Science for Life Laboratory, Uppsala University, Uppsala, Sweden; 4 Department of Cell and Molecular Biology, National Bioinformatics Infrastructure Sweden, Science for Life Laboratory, Uppsala University, Uppsala, Sweden; 5 Department of Organismal Biology—Systematic Biology, Uppsala University, Uppsala, Sweden; 6 Department of Biology, Appalachian State University, Boone, NC, USA; 7 U.S. Department of Agriculture, Agriculture Research Service, Charleston, SC, USA; 8 School of Biological Sciences, University of East Anglia, Norwich Research Park, Norwich, United Kingdom; 9 Biological Sciences Department, Webster University, St. Louis, MO, USA

**Keywords:** speciation, gene flow, population genomics, bumblebees, islands of divergence

## Abstract

Evidence is accumulating that gene flow commonly occurs between recently diverged species, despite the existence of barriers to gene flow in their genomes. However, we still know little about what regions of the genome become barriers to gene flow and how such barriers form. Here, we compare genetic differentiation across the genomes of bumblebee species living in sympatry and allopatry to reveal the potential impact of gene flow during species divergence and uncover genetic barrier loci. We first compared the genomes of the alpine bumblebee *Bombus sylvicola* and a previously unidentified sister species living in sympatry in the Rocky Mountains, revealing prominent islands of elevated genetic divergence in the genome that colocalize with centromeres and regions of low recombination. This same pattern is observed between the genomes of another pair of closely related species living in allopatry (*B. bifarius* and *B. vancouverensis*). Strikingly however, the genomic islands exhibit significantly elevated absolute divergence (*d*_XY_) in the sympatric, but not the allopatric, comparison indicating that they contain loci that have acted as barriers to historical gene flow in sympatry. Our results suggest that intrinsic barriers to gene flow between species may often accumulate in regions of low recombination and near centromeres through processes such as genetic hitchhiking, and that divergence in these regions is accentuated in the presence of gene flow.

## Introduction

Genome-wide comparisons of genetic variation between species provide information about their history of divergence from a common ancestor. As populations diverge, barriers to gene flow eventually arise at multiple loci in their genomes (termed barrier loci), which contain variants that govern ecological specialization or generate intrinsic genomic incompatibilities (Ravinet et al. 2017). Such barriers to gene flow may accumulate while gene flow is ongoing, such as in the case of sympatric or parapatric speciation, or alternatively in the absence of gene flow according to a strict allopatric model (Coyne and Orr 2004). Periods of gene flow can also occur when there is secondary contact between diverging species, during which barriers to introgression may either accumulate or break down (Kirkpatrick and Ravigné 2002; Rundle and Nosil 2005). When species hybridize, selection is predicted to act against gene flow at barrier loci but not in the rest of the genome (Wu 2001). However, despite intense study of many systems, we still lack a general understanding of which genomic regions tend to harbor barrier loci, how such barriers accumulate, and how the transition from incomplete to complete reproductive isolation occurs. 

Comparisons of the genomes of closely related species often reveal a heterogeneous landscape of divergence, which contain distinct peaks that have been described as islands of divergence (IoDs) (Turner et al. 2005). This pattern has been interpreted according to several models. Firstly, if gene flow has been common between the species either during initial divergence in sympatry or after secondary contact, then IoDs could represent barrier loci where introgression is disadvantageous and selected against, leading to increased levels of divergence (Wu 2001). A large number of studies have used comparisons of genome-wide variation in recently diverged species in order to identify IoDs, often with the aim of revealing genes that promote local adaptation and/or speciation and are recalcitrant to gene flow according to this model (Turner et al. 2005; Ellegren et al. 2012; Martin et al. 2013; Renaut et al. 2013; Poelstra et al. 2014; Soria-Carrasco et al. 2014; Lamichhaney et al. 2015; Malinsky et al. 2015; Chapman et al. 2016; Talla et al. 2017; Irwin et al. 2018; Papadopulos et al. 2019; Stankowski et al. 2019).

Secondly, in some cases, it has been shown that IoDs represent ancient balanced polymorphisms that segregated in the ancestral populations (Guerrero and Hahn 2017; Han et al. 2017). Islands of divergence formed by this model are also expected to contain loci of adaptive significance. Such loci evolve under balancing selection in the ancestral population followed by sorting of divergent ancient haplotypes in the descendent populations.

Thirdly, IoDs with elevated relative divergence can form in the absence of gene flow via linked selection due to genetic hitchhiking or background selection, which has a greater effect in regions of reduced recombination (Nordborg et al. 1996; Charlesworth et al. 1997; Turner and Hahn 2010; Cruickshank and Hahn 2014). This process results in elevated levels of relative divergence (*F*_ST_) in regions of low recombination between recently diverged species that have not experienced gene flow. This can result in the formation of IoDs in regions of low recombination, termed “incidental islands,” which do not harbor barrier loci or have a function in adaptation or speciation.

In some species comparisons, IoDs have been identified that are clearly associated with ecological specializations, such as beaks of Darwin’s finches (Lamichhaney et al. 2015; Han et al. 2017), or known incompatibilities, such as generation of melanoma in hybrids of swordtail fish (Powell et al. 2020). However, many studies have identified landscapes of divergence more consistent with the incidental island model, in which IoDs tend to occur in regions of low recombination (Turner and Hahn 2010; Cruickshank and Hahn 2014; Burri et al. 2015; Feulner et al. 2015; Ravinet et al. 2017; Talla et al. 2017) and may not be relevant for adaptation or speciation. It is however important to note that these processes are not mutually exclusive: IoDs formed due to linked selection or balancing selection could also harbor barrier loci (Ravinet et al. 2017) and the landscape of divergence could be shaped by multiple interacting processes (Chapman et al. 2016; Papadopulos et al. 2019). Furthermore, there is also evidence from species that are known to hybridize that the rate of gene flow is correlated with recombination rate, which suggests that identification of IoDs in regions of low recombination does not preclude them from containing barriers to introgression (Schumer et al. 2018; Martin et al. 2019).

A key difference predicted between IoDs formed under these models is that those that have acted as barriers to gene flow or that are involved in ancient balanced polymorphisms should have longer coalescence times than the rest of the genome, resulting in IoDs with elevated absolute divergence measured by *d*_XY_. Conversely, IoDs formed by ongoing linked selection in the absence of gene flow should not have elevated *d*_XY_ and may even have shorter coalescence times than the rest of the genome, due to these processes also occurring in the ancestral population, which results in reduced *d*_XY_ (Cruickshank and Hahn 2014; Irwin et al. 2018). Hence, the genomic landscape of absolute divergence reflects the presence or absence of historical gene flow or ancient balanced polymorphisms, and the existence of barrier loci. This landscape can be interrogated to learn about the processes that have occurred as species diverged.

Previous studies have demonstrated that regions of low recombination, particularly in the vicinity of centromeres, tend to accumulate elevated relative divergence in the absence of gene flow, due to the effects of linked selection (Nachman and Payseur 2012; Roesti et al. 2012). However, it is unclear whether this process also leads to the accumulation of barriers to gene flow in these regions, which could be important in establishing reproductive isolation. In addition, some models of divergence with gene flow where selection acts on many loci have also been shown to result in elevated divergence in regions of low recombination (Michel et al. 2010). Comparisons of patterns of divergence in pairs of species that have experienced different degrees of gene flow during their divergence can help reveal whether divergence in regions of low recombination is associated with the presence of barriers to gene flow.

Here, we use population-scale genome sequencing to infer the mechanisms behind species divergence within the bumblebee subgenus *Pyrobombus* (Hines et al. 2006; Cameron et al. 2007; Martinet et al. 2019). Convergence in coloration due to Müllerian mimicry results in highly similar morphologies among bumblebee species (Williams 2007; Ezray et al. 2019), which rely mainly on chemical signaling for mate recognition (Goulson 2003). These factors make species recognition particularly difficult in bumblebees and studies utilizing multiple genetic loci have recently resulted in the discovery of several previously undescribed species (Martinet et al. 2019; Ghisbain et al. 2020). The number of bumblebee species is likely underestimated with many cryptic species living in sympatry, which may have experienced gene flow during their formation (Bertsch et al. 2004; Murray et al. 2008; Bossert 2015).

We constructed a highly contiguous genome assembly of the species *Bombus sylvicola*, and surveyed genomic variation in this species by whole-genome resequencing 284 samples from across the Rocky Mountains in Colorado. Unexpectedly, these samples fell into two distinct genetic clusters, revealing the presence of a previously unknown cryptic species living in sympatry with *B. sylvicola*, which we name *B. incognitus*. We performed genome-wide comparisons between these two sympatric species and contrasted them with genomic divergence between another pair of closely related species living mainly in allopatry (*B. bifarius* and *B. vancouverensis*) to uncover evidence for historical gene flow and identify and characterize regions of the genome that have likely acted as barriers to gene flow in the past. Analysis of the genomic landscape of divergence reveals signals of gene flow in the sympatric but not the allopatric pair, and provides important insights into how genomic architecture influences the formation of barrier loci.

## Results

### A Highly Contiguous Genome Assembly of *Bombus sylvicola*

We used a combination of Oxford Nanopore (ONT) and 10× Chromium sequencing to generate a genome assembly of the bumblebee *B. sylvicola* using a single haploid drone sample for each technology (see Materials and Methods). A recent study analyzed genetic and morphological differentiation between *B. sylvicola* collected in northern Alaska and *B. lapponicus* collected in northern Sweden (Martinet et al. 2019). Based on relatively low levels of divergence, this study redefined *B. sylvicola* as the subspecies *B. lapponicus sylvicola.* However, here, we maintain the previous name *B. sylvicola* for our samples for consistency with previous ecological studies in this region and because their relationship to the populations in Alaska has not been directly tested. The sequencing and assembly resulted in a set of 592 contigs with a total length of 252,081,862 bp and an N50 of 3,020,754 bp. Analysis of genome completeness estimated that 97.9% (5,865) of BUSCO genes were complete in the assembly and only 1.5% (92 genes) were undetected. We estimated the position and orientation of contigs on chromosomes via a whole-genome alignment with the *B. terrestris* genome. This resulted in the preliminary placement of 91.1% of the *B. sylvicola* genome onto 18 likely chromosomes (hereafter, pseudochromosomes). Our annotation pipeline annotated 11,585 genes across the *B. sylvicola* genome (14% of the genome is found in exons). This is highly comparable to the 11,874 genes in the *B. terrestris* gene set (v. 1.0) and the 12,728 genes in the *B. impatiens* gene set (v. 2.1).

We analyzed both the raw ONT reads and the assembled contigs to identify putative centromere-associated sequences (see Materials and Methods). This analysis revealed the presence of three 15-bp monomers at high frequency in the ONT reads, each differing by 1–2 bp and forming tandem repeat arrays (supplementary fig. S1, Supplementary Material online). Repeat arrays occur near or at contig boundaries, indicating that the true arrays are likely longer and that the assembler has failed to assemble them further due to their repeat nature (Kolmogorov et al. 2019). The prevalence and location in the vicinity of assembly gaps suggest that the repeats occur near centromeres. We find 83 occurrences of the tandem repeat array in lengths ranging from 28 to 22,416 bp (mean length = 1,911 bp) across the genome, with 48 occurrences across 15 of the 18 pseudochromosomes (the remaining 35 are on unplaced contigs). Nine of the 18 pseudochromosomes contain arrays greater than 1 kb in length and, in most cases, arrays occur in one region per pseudochromosome, indicating the likely locations of centromeres.

### Population-Scale Sequencing Leads to Identification of a New Species

**Fig. 1.**
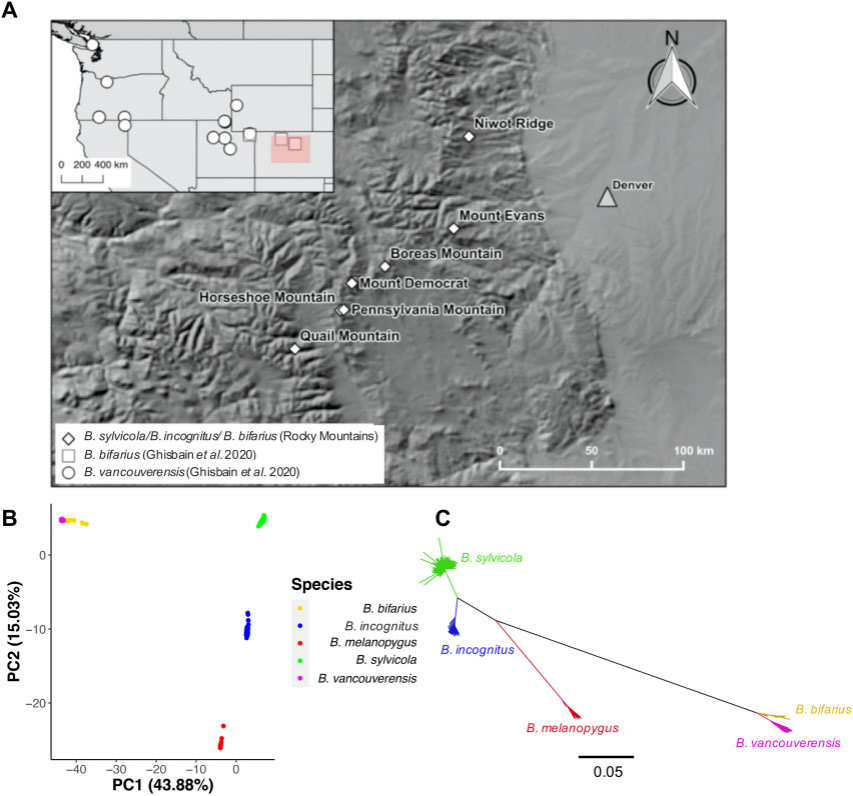
Sampling locations and genomic variation in *Pyrobombus* bumblebees. (*A*) Map showing the seven locations in Colorado where *Pyrobombus* bees were sampled for this study, as well as the sampling locations of *Bombus bifarius* and *B. vancouverensis* from a previous study (inset) (Ghisbain et al. 2020). *Bombus melanopygus* was collected widely across western USA in a previous study (Tian et al. 2019). (*B*) Principal component analysis and (*C*) a neighbor-joining tree based on genome-wide SNPs thinned for one SNP every 10 kb of the five *Pyrobombus* species included in this study revealed distinct genetic divergence between *B. sylvicola* and *B. incognitus*. Scale bar on tree represents sequence divergence (%).

We collected 284 female worker bees identified phenotypically as *B. sylvicola* and 17 identified as *B. bifarius* from seven localities in the Rocky Mountains, Colorado (fig. 1*A*). We obtained Illumina whole-genome sequencing (WGS) data for all samples. We also obtained published WGS data from four samples of *B. bifarius* and 17 samples of *B. vancouverensis* collected from Colorado across north-eastern USA (Ghisbain et al. 2020) and 21 samples of *B. melanopygus* from western USA (Tian et al. 2019) giving a total of 343 resequenced genomes of bumblebees within the *Pyrobombus* subgenus (fig. 1*A*). We mapped these WGS data sets to our *B. sylvicola* genome assembly and performed variant calling. The mean coverage across all samples was 14.7× and we inferred 15,094,475 SNPs (see supplementary tables S1 and S2, Supplementary Material online, for full details of all samples).

A principal components analysis (PCA) of the genome-wide SNP data set showed clear clustering by species (fig. 1*B*). Surprisingly, the 284 samples identified as *B. sylvicola* were split into two distinct clusters, containing 217 and 67 samples, respectively, with no observations of intermediates between the two clusters. The *B. bifarius* and *B. vancouverensis* samples also formed two distinct clusters, consistent with their assignment as two separate species by Ghisbain et al. (2020). A neighbor-joining tree also strongly supported the division of the *B. sylvicola* samples into two clusters with the *B. bifarius–B. vancouverensis* pair placed distantly from these clusters (fig. 1*C*). We also generated a neighbor-net network based on SNPs across the genome to check for any conflicting signals or alternative phylogenetic histories (supplementary fig. S2, Supplementary Material online), which demonstrates that the underlying evolutionary history of these species is treelike. Taken together, these data indicate the presence of a cryptic species within the purported *B. sylvicola* samples.

We next attempted to reveal the identity of this cryptic species. *Bombus melanopygus* appears as an outgroup to the two *B. sylvicola-*like clusters, placing the cryptic species within the *B. lapponicus–B. sylvicola–B. monticola* species complex (Cameron et al. 2007; Williams et al. 2014; Martinet et al. 2019). Martinet et al. (2019) used both morphological and genetic analysis to delineate relationships of all species within this complex and identified a previously undescribed cryptic species with similar morphology to *B. sylvicola*, which they call *B. interacti*, which could potentially match the unexpected cluster we identified. In order to test this possibility, we compared sequences of our samples at the PEPCK and COI loci with those of all species in the complex presented in Martinet *et al.* (2019).

We determined the PEPCK sequences of all of our samples from the WGS data (supplementary fig. S3*A*, Supplementary Material online) and found that samples from our larger *B. sylvicola*-like cluster closely corresponded to the *B. sylvicola* and *B. lapponicus* samples from Martinet et al. (2019), with three samples matching exactly. However, our smaller *B. sylvicola*-like cluster did not show similarity with any other species examined. It is most similar to *B. sylvicola* and clearly distinct from *B. interacti.* We next generated COI sequences from a subset of our samples using PCR and Sanger sequencing (supplementary fig. S3*B*, Supplementary Material online). Here, we find a similar pattern, where the samples from our larger *B. sylvicola*-like cluster again correspond most closely with the *B. sylvicola* and *B. lapponicus* samples from Martinet et al. (2019), whereas samples from the smaller *B. sylvicola*-like cluster are closely related but distinct from these samples, and the *B. interacti* samples are more distant. Taken together, these results suggest that the samples from the larger *B. sylvicola*-like cluster in our data set correspond to samples identified as *B. sylvicola* in previous studies, whereas the samples in the smaller *B. sylvicola-*like cluster represent a previously undescribed species. We assign this species the provisional name *Bombus incognitus.*

We performed a detailed characterization of the anatomical structures on the heads and abdomens of a subset of samples identified genetically as *B. sylvicola* and *B. incognitus* (see Materials and Methods and supplementary table S3, Supplementary Material online). None of these traits could be used to distinguish between the two species. *Bombus sylvicola* samples were significantly larger on average based on measurements of intertegular distance, which is a proxy of body size (mean = 3.84 mm and 3.59 mm for *B. sylvicola* and *B. incognitus*, respectively; Wilcoxon rank sum test, *W* = 7673, *P* = 1.304 × 10^−5^**;**supplementary fig. S4, Supplementary Material online). The locations where samples of each species were found showed substantial overlap (supplementary table S2, Supplementary Material online). Both species were collected together on six out of the seven localities (*B. incognitus* was not present among the 28 samples collected on Mount Democrat). The two species were found at overlapping elevations, although there was a significant tendency for *B. incognitus* to be found at lower elevations (mean elevations 3,792 and 3,664 m for *B. sylvicola* and *B. incognitus*, respectively; Wilcoxon rank sum test, *W* = 11192, *P* = 7.05 × 10^−12^, supplementary fig. S4, Supplementary Material online). In summary, *B. sylvicola* and *B. incognitus* are cryptic species found in sympatry across our sampling localities.

### The Genomic Landscape of Divergence Differs between Sympatric and Allopatric Species Pairs

We used our data set to compare genome divergence on multiple spatial and temporal scales. Firstly, the two most geographically and genetically distant (as revealed by a PCA of all *B. sylvicola* samples; supplementary fig. S5, Supplementary Material online) populations of *B. sylvicola* that we sampled were from Niwot Ridge and Quail Mountain, Colorado (hereafter, within-species pair). Secondly, we sampled two closely related species existing in sympatry across this range (*B. sylvicola* and *B. incognitus*; hereafter, sympatric pair), which allowed us to investigate genome divergence in species that may have had the opportunity to undergo gene flow in the past. Thirdly, we used genomic data for two other closely related Pyrobombus species that exist mainly in allopatry, (*B. bifarius* and *B. vancouverensis*; hereafter, allopatric pair), which we hypothesize underwent speciation in the absence of gene flow. These species are separated geographically with only a narrow range of overlap in mountains in Utah, with no evidence of hybridization (Ghisbain et al. 2020).

**Fig. 2.**
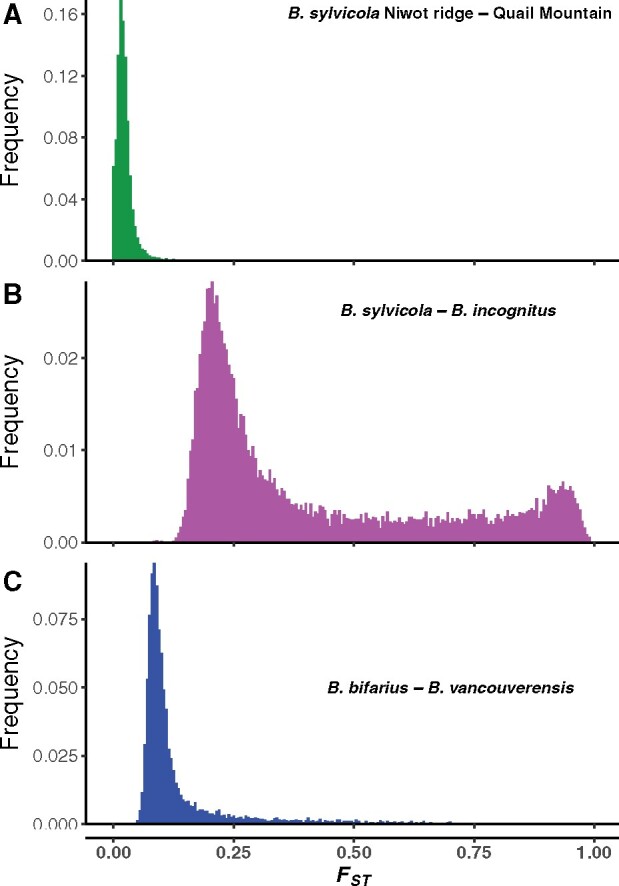
Histograms showing distributions of *F*_ST_ measured in 20-kb windows across the genome for (*A*) a within-species comparison of two *Bombus sylvicola* populations, Niwot Ridge (*n* = 43) and Quail Mountain (*n* = 17), (*B*) a sympatric comparison of *B. sylvicola* (*n* = 217), and *B. incognitus* (*n* = 67), and (*C*) an allopatric comparison of *B. bifarius* (*n* = 21), and *B. vancouverensis* (*n* = 17). Note the distinct bimodal distribution of the sympatric comparison, indicating differential divergence across the genome.

In order to compare landscapes of divergence at these different scales, we carried out genome-wide sliding-window *F*_ST_ scans. Average genome-wide *F*_ST_ was 0.02, 0.41, and 0.14 for the within-species, sympatric, and allopatric pairs, respectively. Both the within-species and allopatric pairs displayed typical *F*_ST_ distributions of a single large peak centered close to the median score with a tail representing relatively few regions with heightened divergence (fig. 2*A* and *C*). However, the sympatric pair displayed a striking bimodal distribution of *F*_ST_, with a large peak centered at 0.21 followed by a second peak of extreme divergence centered at 0.93 (fig. 2*B*). The sympatric pair therefore stands out as having a distinct portion of the genome with highly elevated divergence. This pattern has been observed for other pairs of species that diverged under conditions of gene flow (Seehausen et al. 2014).

We used estimates of genetic variation and divergence to estimate the effective population size and timings of the splits between species (table 1). *Bombus incognitus* showed the highest levels of genetic variation despite being less abundant than *B. sylvicola* in our sampling localities, and both of these species exhibited higher levels of variation than *B. bifarius* and *B. vancouverensis*. Estimates of *N*_e_ are in the same range as estimated for honeybees (Wallberg et al. 2014). Average *F*_ST_ between *B. sylvicola* and *B. incognitus* based on all SNPs located outside of regions of extreme divergence was 0.34. This translates into an estimated divergence time of *t *=* *396,000 (95% CI 389,000–403,000) generations since the species split under a simple demographic model using estimates for *θ*_w_ per base and *N*_e_ (table 1). For the allopatric pair, average *F*_ST_ is 0.12, which indicates a divergence time of *t *=* *67,290 (95% CI 66,177–68,514) generations. Given the generation time of bumblebees is one generation per year, these divergence times translate directly into years.

**Table 1.** Summary Statistics for the Four *Pyrobombus* Species.

Species	*N*	Number of Chromosomes	Number of SNPs	Effective PopulationSize, *N*_e_	Watterson’s ThetaPer Base (*θ*_w_)	NucleotideDiversity (*π*)
*Bombus sylvicola*	217	434	4,655,117	260,000	0.0028	0.0025
*Bombus incognitus*	67	134	4,891,459	320,000	0.0035	0.0028
*Bombus bifarius*	21	41	1,924,407	164,000	0.0018	0.0014
*Bombus vancouverensis*	17	18	2,057,379	216,000	0.0023	0.0016

### Genomic Islands of Divergence Evolve in Similar Locations in Independent Species Comparisons

**Fig. 3.**
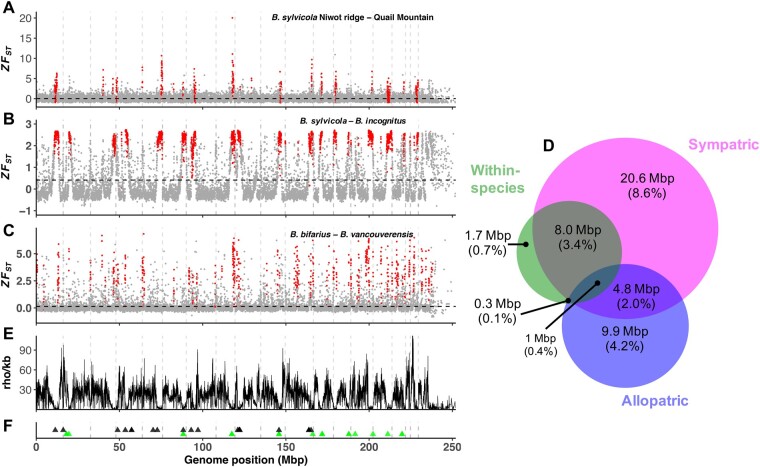
Genome-wide ZF_ST_ scores measured in 20-kb nonoverlapping sliding windows for (*A*) within-species *Bombus sylvicola* Niwot Ridge–Quail Mountain comparison, (*B*) sympatric *B. sylvicola*–*B. incognitus* comparison, and (*C*) allopatric *B. bifarius*–*B. vancouverensis* comparison. Red dots represent 20-kb windows that are located in islands of divergence (IoDs), defined as regions of extreme divergence (ZF_ST_ scores >2) over 100 kb in length. Horizontal black dashed lines represent mean values. (*D*) Venn diagram showing overlap in positions of IoDs between comparisons, including size of overlap in Mb and as a percentage of the genome. (*E*) Recombination rate variation across the genome measured in ρ/kb. (*F*) Positions of putative centromere tandem repeat arrays, where black triangles indicate arrays >1 kb and green indicates arrays <1 kb. Vertical gray dashed lines represent boundaries of the 18 pseudochromosomes, with data from unplaced contigs shown to the right of the last pseudochromosome.

We converted *F*_ST_ values to *Z*-scores (ZF_ST_; fig. 3*A–C*) and used them to define “highly divergent windows” in each comparison, where ZF_ST_ ≥2 (2 or more SDs above the median *F*_ST_). This resulted in 486 highly divergent windows (3.86% of the genome) for the within-species pair, 1,758 windows (13.95% of the genome) for the sympatric pair, and 842 windows (6.68% of the genome) for the allopatric pair. It is strikingly apparent from the genome-wide ZF_ST_ plots (fig. 3*A–C*), particularly for the sympatric pair (fig. 3*B*), that genome variation contains several large blocks of extreme divergence. We defined blocks larger than 100 kb as IoDs (see Materials and Methods). Using this definition, there are 20 IoDs in the within-species pair (fig. 3*A*; average length of 560 kb), 28 IoDs in the sympatric pair (fig. 3*B*; average length of 1.26 Mb), and 68 IoDs in the allopatric pair (fig. 3*C*; average length of 223 kb). The longest 18 IoDs in the sympatric pair were positioned over 17 pseudochromosomes, demonstrating that, in the majority of cases, pseudochromosomes contain a single major IoD in sympatry (fig. 3*B*). A similar pattern is observed in the within-species and allopatric pairs, although IoDs are smaller and not found on all pseudochromosomes. The sympatric pair is therefore distinguished by the presence of one large IoD per pseudochromosome and having the greatest proportion of the genome in IoDs.

There was a highly significant overlap in the location of IoDs between the independent population/species comparisons, assessed using permutation tests (fig. 3*D* and supplementary fig. S6, Supplementary Material online). Notably, 75% of within-species IoDs overlapped with a sympatric IoD (permutation test, *Z*-score = 5.194, *P* = 0.001; supplementary fig. S6*A*, Supplementary Material online) and 43% of allopatric IoDs overlapped with a sympatric IoD (permutation test, *Z*-score = 4.567, *P* = 0.001; supplementary fig. S6*B*, Supplementary Material online). The within-species and allopatric comparisons showed some overlap but it was not significant at *P* = 0.05 (permutation test, *Z*-score = 1.576, *P* = 0.09; supplementary fig. S6*C*, Supplementary Material online). The observation of IoDs in the within-species comparison shows that divergence can accumulate in these regions in the absence of reproductive isolation.

### Islands of Divergence Are Associated with Reduced Recombination Rate and a Common Satellite Repeat

We estimated genome-wide variation in recombination rate in *B. sylvicola* using LDhat (fig. 3*E*) (McVean et al. 2004). We found a significant positive correlation between recombination rate and GC content (Pearson’s *r *=* *0.23, *P* < 0.001) which has been found previously in bumblebees (Kawakami et al. 2019) and is observed widely in sexual eukaryotes (likely as a result of GC-biased gene conversion in high-recombining regions) (Pessia et al. 2012). We found a strongly significant negative correlation between ZF_ST_ and recombination rate that was particularly pronounced in the sympatric pair (Spearman’s rho = −0.78, *P* < 2.2 × 10^−16^) but lower in the within-species and allopatric pairs (within-species: Spearman’s rho = −0.02, *P* = 0.03; allopatric: Spearman’s rho = −0.24, *P* < 2.2 × 10^−16^). In addition, we found significantly reduced recombination rates in IoDs in all species pairs (Wilcoxon rank sum test, *P* < 2.2 × 10^−16^ in all cases; supplementary figs. S7 and S8, Supplementary Material online). This finding is less pronounced in the allopatric pair, where regions of high divergence are found to occur in regions with higher recombination rates. We note however that as the method we employed estimates recombination rates based on patterns of genetic variation it is likely that the extremely low levels of nucleotide diversity in the IoDs in *B. sylvicola* result in lower accuracy to measure recombination rates in these regions.

In all three comparisons, we found significantly lower GC content, lower mappability (a measure of sequence uniqueness in the genome), and higher repeat content inside IoDs compared with the rest of the genome (Wilcoxon rank sum tests, all significant at *P* < 2.2 × 10^−16^; table 2; and supplementary fig. S7, Supplementary Material online). These observations are consistent with a tendency for IoDs to occur in regions of low recombination. We also observed significantly greater gene density inside IoDs compared with random expectations in the allopatric comparison (table 2). In the sympatric comparison, IoDs comprise 13.9% of the genome and contain 2,135 genes (19.8%). It is plausible that the high levels of divergence across this large number of genes in IoDs have functional consequences that could contribute to adaptation or intrinsic barriers to gene flow.

**Table 2.** Differences in Genomic Content Inside and Outside of IoDs.

Metric	Recombination Rate (ρ/kb)		GC Content (%)		Repeat Content (%)		Mappability		Exonic Sequence (%)	
IoDs	In	Out	In	Out	In	Out	In	Out	In	Out
Within-species	2.3***	21.7	36.6***	38.1	21.0***	12.2	0.97***	0.99	13.4	15.7
Sympatric	2.7***	23.8	36.4***	38.3	16.1***	12.0	0.97***	0.99	15.8	15.5
Allopatric	11.1***	21.5	34.4***	38.3	18.4***	12.1	0.97***	0.99	29.0***	14.6

Note.—

*** *P* < 0.001.

Significance of difference in recombination rate, proportion GC content, repeat content, and mappability inside and outside of IoDs assessed with Wilcoxon rank sum test. Significance of proportion of exonic sequence inside of IoDs assessed using permutation tests.

We next tested for associations between the locations of the likely centromeric tandem repeat arrays and the locations of IoDs in each population/species comparison (fig. 3*F* and supplementary fig. S9, Supplementary Material online). There is a particularly strong association in the sympatric pair, where we observe one dominant large IoD (>100 kb) per pseudochromosome. We found a significant overlap of 36 (75%) repeats overlapping with 13 sympatric IoDs, which was 4.6× greater than expected by chance (permutation test, *Z*-score = 6.33, *P* = 0.001). Significant overlap was also observed in the within-species comparison, where 12 (25%) repeats overlapped with four IoDs (permutation test, *Z*-score = 3.25, *P* = 0.02). In the allopatric pair, there was overlap between IoDs and repeats with eight (17%) repeats overlapping six IoDs, but this was not significantly different from the overlap expected by chance (permutation test, *Z*-score = 2.16, *P* = 0.06). Hence, although there is a tendency for IoDs to occur near centromeres in all comparisons, there is a particularly strong association in the sympatric comparison. It is unlikely that errors in read mapping or variant calling in repetitive regions contributed to the elevated divergence inferred in IoDs. Inspection of IoDs reveals that elevated divergence occurs both in repetitive regions and in flanking nonrepetitive regions (supplementary fig. S10, Supplementary Material online).

In order to test whether IoDs may represent large structural inversions, we ran the program manta (Chen et al. 2016) on a subset of the *B. sylvicola* and *B. incognitus* bam files. We found evidence for only three short inversions that were fixed between the two species: one of 6,351 bp on contig_013, one of 3,059 bp on contig_026, and one of 1,691 bp on contig_118. All three of these putative inversions are found within IoDs and likely lead to some of the high divergence we see in these regions, however they make up only a small fraction of the IoDs they are found in. We therefore did not find support for any of the identified IoDs representing structural rearrangements.

### Genomic Islands of Divergence Have Elevated *d*_XY_ in the Sympatric but Not the Allopatric Pair

**Fig. 4.**
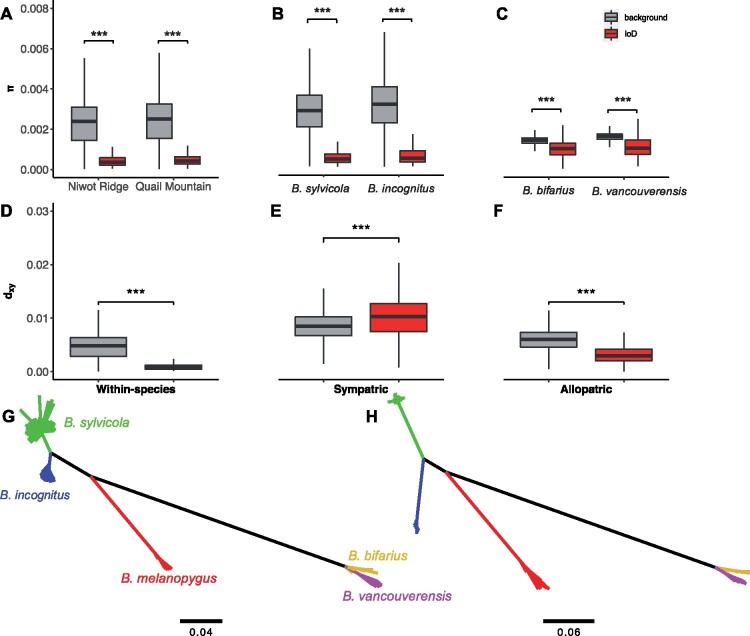
Differences in nucleotide diversity (*π*) and absolute divergence (*d*_XY_) inside and outside of islands of divergence (IoDs, where ZF_ST_ >2) for (*A* and *D*) a within-species comparison of two *Bombus sylvicola* populations (Niwot Ridge and Quail Mountain), (*B* and *E*) a sympatric comparison of *B. sylvicola* and *B. incognitus*, and (*C* and *F*) an allopatric comparison of *B. bifarius* and *B. vancouverensis*. Neighbor-joining trees based on single nucleotide polymorphisms (*G*) outside of IoDs and (*H*) inside IoDs in the sympatric comparison. Scale bars on trees represent sequence divergence (%).

For all species/population comparisons, nucleotide diversity (*π*) was significantly lower in IoDs compared with the rest of the genome (Wilcoxon rank sum test, *P* < 2.2 × 10^−16^ in all cases; fig. 4*A–C*; supplementary figs. S11–S13, Supplementary Material online). This is consistent with the action of linked selection (background selection and/or genetic hitchhiking) on these regions, which both increases relative divergence and decreases levels of genetic variation. There is a more pronounced reduction of *π* in IoDs in both sympatric species (80% and 77% decrease in *π* inside compared with outside IoDs in *B. sylvicola* and *B. incognitus*, respectively) compared with the allopatric species (28% and 30% decrease in *π* inside compared with outside IoDs in *B. bifarius* and *B. vancouverensis*, respectively). IoDs are therefore more extensive and exhibit lower within-species variation in the sympatric comparison.

We found *d*_XY_ to be significantly lower in IoDs compared with the rest of the genome in the within-species and allopatric pairs (Wilcoxon rank sum tests, *P* < 2.2 × 10^−16^ in both cases), with a 66% and 48% reduction in *d*_XY_ inside IoDs, respectively. Yet intriguingly, we found *d*_XY_ to be significantly elevated inside compared with outside IoDs in the sympatric pair (Wilcoxon rank sum test, *P* < 2.2 × 10^−16^), with a 17% increase inside IoDs (fig. 4*D–F*). Neighbor-joining trees based on only SNPs found 1) within IoDs and 2) outside of IoDs revealed the same topology, but substantially longer branch lengths in the sympatric pair within IoDs, consistent with their elevated *d*_XY_ (fig. 4*G* and *H*). The sympatric comparison is therefore distinguished by a strikingly bimodal distribution of *F*_ST_ across the genome, and IoDs with significantly elevated *d*_XY_.

Two main evolutionary scenarios have been demonstrated to result in elevated *d*_XY_ in IoDs. The first is divergence with differential gene flow (Cruickshank and Hahn 2014). The second is the presence of ancient balanced polymorphisms in the ancestral population that are sorted in the descendent populations (Guerrero and Hahn 2017). However, the association of IoDs with elevated *d*_XY_ in the sympatric but not the allopatric comparison is most parsimoniously explained by differences in the incidence of gene flow between the two comparisons, suggesting that the sympatric IoDs have been shaped by differential gene flow (see Discussion). The observation of reduced *d*_XY_ in IoDs in the allopatric pair is most consistent with ongoing linked selection resulting in “incidental islands.”

Differences in mutation rate or average levels of evolutionary constraint in IoDs compared with the rest of the genome could also potentially influence the differences in *d*_XY_ we observed in these regions. Such differences would generate variation in *d*_XY_ in more distant species comparisons. In order to assess this possibility, we estimated *d*_XY_ in the IoD regions defined by the sympatric comparison in comparisons of both *B. sylvicola* and *B. incognitus* to *B. bifarius* and to a single sample of *B. balteatus*, which belongs to a separate subgenus. In all comparisons, *d*_XY_ inside and outside of IoDs did not significantly differ (Wilcoxon rank sum test, *P* > 0.05; supplementary fig. S14, Supplementary Material online). This indicates that the average rate of nucleotide substitution in IoDs is similar to the rest of the genome.

### Islands of Divergence Are Associated with Extended Drops in Levels of Genetic Variation in the Sympatric Comparison

We calculated the population branch statistic (PBS) (Yi et al. 2010) to assess the relative amount of divergence that had occurred along each branch. PBS correlated strongly between branches in the sympatric pair (Spearman’s *rho* = 0.62, *P* < 2.2 × 10^−16^) as well as with *F*_ST_ in both comparisons (Spearman’s *rho* = 0.85 and 0.60 in the sympatric and allopatric comparisons, respectively; *P* < 2.2 × 10^−16^ in both comparisons). As expected, IoDs have significantly greater PBS compared with the rest of the genome in all four species (Wilcoxon rank sum test, *P* < 2.2 × 10^−16^ in all cases), showing there to be strong correspondence between the locations of regions of elevated divergence that have formed on both branches leading from the common ancestor in both species pairs (supplementary fig. S15, Supplementary Material online).

**Fig. 5.**
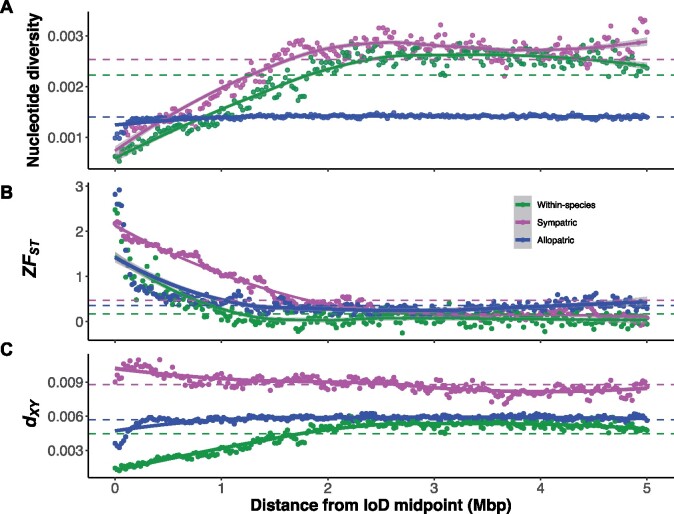
Average changes in (*A*) nucleotide diversity (*π*), (*B*) relative divergence (ZF_ST_), and (*C*) absolute divergence (*d*_XY_) stepping away from centers of Islands of Divergence in 20-kb windows in both directions. Within-species comparisons are Niwot ridge and Quail Mountain populations of *Bombus sylvicola*, the sympatric comparison is *B. sylvicola* and *B. incognitus*, and the allopatric comparison is *B. bifarius* and *B. vancouverensis.* For nucleotide diversity, data for only one species from each pair are shown for clarity: Within-species = Niwot Ridge, sympatric = *B. sylvicola*, allopatric = *B. bifarius.* Dashed lines represent mean values. Smooth curves are based on locally estimated scatterplot smoothing (LOESS), with 95% confidence intervals shown in gray.

To further characterize IoDs between our pairs of species, we calculated average *π*, ZF_ST_, and *d*_XY_ with increasing distance from their midpoints (fig. 5). There is a more extensive reduction of *π* in IoDs in the sympatric pair compared with the allopatric pair (fig. 5*A*). For the sympatric species, average *π* values remained below the genome average up to ∼1.5 Mb away from the center of the IoDs, however, for the allopatric species, this distance was ∼0.2 Mb. A more extensive drop in within-species variation at IoDs in the sympatric pair is unlikely to be explained by a greater effect of linked selection and suggests the influence of differential gene flow.

Average ZF_ST_ values reflected the larger size of IoDs in the sympatric pair compared with the within-species and allopatric pairs: in the sympatric pair, ZF_ST_ did not return to the genome average until ∼1.8 Mb away from the center of the IoDs, whereas for the within-species pair and allopatric pair the distance was ∼1 Mb (fig. 5*B*). There is a stark contrast in patterns of *d*_XY_ in IoDs in the sympatric compared with the within-species and allopatric pairs (fig. 5*C*). For the sympatric pair, average *d*_XY_ values remained “above” the genome average up to ∼2.6 Mb away from IoD centers, whereas for the within-species and allopatric pairs *d*_XY_ values remained “below” the genome average until ∼1.8 and 0.4 Mb away from IoD centers, respectively. IoDs in the sympatric comparison are therefore larger, show more extensive reductions in genetic variation, and are distinguished by elevated absolute divergence (*d*_XY_). These observations are all consistent with a scenario where IoDs in the sympatric comparison have acted as barriers against historic gene flow. In contrast, the lower *d*_XY_ in IoDs in the within-species and allopatric pairs likely reflect the action of linked selection in the ancestral populations, which reduces coalescence times between species (Irwin et al. 2018).

## Discussion

We analyzed genome variation in multiple closely related species of bumblebees in the *Pyrobombus* subgenus to uncover mechanisms of species divergence and isolation. Analysis of 284 specimens classified as *B. sylvicola* in the Rocky Mountains revealed a previously undetected cryptic species living in sympatry, which we call *B. incognitus.* Genome-wide comparisons of genetic variation between *B. sylvicola* and *B. incognitus* revealed a striking bimodal landscape of divergence, with extensive pronounced genomic IoDs in the vicinity of centromeres. Our analysis indicates that these centromere-associated IoDs contain barrier loci that have restricted gene flow in these regions between *B. sylvicola* and *B. incognitus* in the past, whereas gene flow was able to continue to a greater extent elsewhere in the genome. We find no evidence of contemporary hybridization between these species, suggesting that current gene flow is rare or nonexistent. Thus, our findings provide a window into the processes that lead to reproductive isolation.

### *Bombus incognitus* Is a Previously Undetected Bumblebee Species

The newly discovered species *B. incognitus* was indistinguishable from *B. sylvicola* using typical diagnostic characters used to identify *B. syvicola*, and all *B. incognitus* samples were initially classified as *B. sylvicola* on the basis of morphology. Analysis of the population genomic data indicated the presence of two distinct clusters with high divergence (*F*_ST_ = 0.41) across most sampling locations, strongly indicating the presence of two species. A more detailed comparison of the head anatomy (see Materials and Methods) did not reveal any characters that distinguish between these two species. However, we observed that the *B. incognitus* we collected were on an average 6.6% smaller than *B. sylvicola*, and were found at lower altitudes despite an overlapping range.

Our whole-genome comparisons demonstrate that *B. sylvicola* and *B. incognitus* are more closely related to each other than either is to *B. melanopygus*. This places *B. incognitus* within a clade of the Pyrobombus subgenus that also contains *B. bimaculatus*, *B. monticola*, *B. konradini*, *B. lapponicus*, and *B. interacti*. *Bombus lapponicus* is an Old-World species with very low divergence in morphology and genetic distance from the New World *B. sylvicola*. Due to this observation, Martinet et al. (2019) suggested that *B. syvicola* should be considered a subspecies of *B. lapponicus* (*B. lapponicus sylvicola*). The species *B. interacti* shows a strong resemblance to *B. sylvicola*. We constructed phylogenies of sequences from the PEPCK and COI loci from our samples compared with samples of all species in the clade presented by (Martinet et al. 2019). Our analyses show a strong correspondence between our *B. sylvicola* samples from Colorado and samples of this species collected in Alaska. However, our *B. incognitus* samples do not cluster together with *B. interacti*, or match any other known species, and are most closely related to the *B. sylvicola* samples. This indicates that *B. incognitus* differs from previously described members of this clade and should be considered a separate species, further adding to the amount of cryptic species diversity recognized in this clade. Further studies are necessary to comprehensively characterize the range and morphology of this species.

We considered the possibility that *B. incognitus* is a hybrid species resulting from interbreeding between *B. sylvicola* and *B. melanopygus* or another more distantly related species. Under this scenario, the majority of the genome, which exhibits low divergence between *B. sylvicola* and *B. incognitus*, would derive from their common ancestor whereas the highly divergent IoDs would have a shorter evolutionary distance to *B. melanopygus* in *B. incognitus*. However, figure 4 shows that this is not the case, as regions defined as IoDs have longer branches throughout the tree and relationships between species are the same within and outside of IoDs. Furthermore, examination of PBS across the genome (supplementary fig. S10, Supplementary Material online) indicates that the level of divergence is similar along the branches leading to *B. sylvicola* and *B. incognitus*, both within and outside of IoDs, indicating that these species diverged from a common ancestor and neither resulted from a hybridization event from a more distantly related species. Finally, there is no indication of hybridization from the neighbor-net network constructed from the data set (supplementary fig. S2, Supplementary Material online).

The lack of intermediates among samples of *B. sylvicola* and *B. incognitus* indicates that the species do not commonly hybridize, although patterns of genomic variation in both species indicate gene flow was ongoing for some time during their divergence. Extensive genome resequencing using field collections could lead to the discovery of more cryptic species in many taxa in future. Such discoveries are arguably more likely in bumblebees because their morphologies often converge due to Müllerian mimicry (Williams 2007; Ezray et al. 2019) and mate recognition occurs mainly via chemical signals (Goulson 2003), which could mask species diversity. Accurate identification of distinct clusters and divergence times requires data from multiple loci or the whole genome due to the effects of incomplete lineage sorting producing conflicting phylogenetic signals (Mallet et al. 2016).

### The Genomic Landscape of Divergence Can Be Shaped by Multiple Factors

Comparisons of genome-wide variation in recently diverged species often reveal a highly variable landscape of divergence containing IoDs (Turner et al. 2005; Ellegren et al. 2012; Renaut et al. 2013; Poelstra et al. 2014; Soria-Carrasco et al. 2014; Lamichhaney et al. 2015; Malinsky et al. 2015; Chapman et al. 2016; Talla et al. 2017; Irwin et al. 2018; Papadopulos et al. 2019; Stankowski et al. 2019; Liu et al. 2020). One interpretation of IoDs is that they contain barrier loci that hinder introgression in species that have experienced gene flow (Wu 2001). Under this scenario, gene flow is prevented at IoDs but continues in the rest of the genome, leading to elevated divergence at IoDs. Two main models of the accumulation of divergence under gene flow have been proposed, which can be viewed as extremes on a continuum (Feder et al. 2012). Under the classical island view, IoDs contain specific outlier loci that form barriers to gene flow. Elevated divergence spreads to nearby tightly linked loci through divergence hitchhiking, whereby variants in linkage with these barrier loci are also prevented from introgressing (Via and West 2008; Via 2012).

Under the second view of divergence under gene flow, termed the continent view of genomic divergence, a much larger number of selected loci contribute to genetic isolation across the genome (Feder et al. 2012). The landscape of divergence is shaped by selection at these loci mediated by the genomic architecture, which includes factors such as linkage relationships, recombination rate variation, and the strength of selection at each locus (Michel et al. 2010; Feder et al. 2012). This second view implies that species barriers are polygenic and does not rely on an effect of divergence hitchhiking. Islands of divergence (or continents) generated by this model are larger and more likely to occur in regions of low recombination, such as inversions (Yeaman 2013) or at centromeres (Turner et al. 2005; Liu et al. 2020) because the effects of linked selection are enhanced in these regions.

It has also been demonstrated by both theoretical and empirical studies that IoDs can evolve in the absence of gene flow. One mechanism that can generate IoDs is sorting of ancient balanced polymorphisms, in which divergent haplotypes segregating in an ancestral population become fixed after the populations split (Guerrero and Hahn 2017). This mechanism likely occurred at loci that govern beak morphology in Darwin’s finches, at which ancient haplotypes that predate the split between species are observed (Han et al. 2017). Another mechanism that can generate IoDs is linked selection (genetic hitchhiking and background selection) mediated by genomic architecture, which causes IoDs to recurrently arise in regions of low recombination (Cruickshank and Hahn 2014; Burri et al. 2015). This phenomenon is connected to the observation across taxa that genetic variation is reduced in regions of low recombination because of linked selection (Begun and Aquadro 1992). This effect causes measures of relative divergence such as *F*_ST_ to be elevated in regions of low recombination. IoDs generated by this effect do not necessarily contain loci involved in adaptation or barriers to gene flow. However, it is possible that IoDs generated by linked selection can harbor-divergent loci that subsequently act as barriers under conditions of gene flow.

### Genomic Barriers to Gene Flow between *B. sylvicola* and *B. incognitus*

Here, we aimed to distinguish between these scenarios and identify putative barrier loci using independent comparisons of genomic variation: 1) between populations of *B. sylvicola* from different localities, 2) between *B. sylvicola* and *B. incognitus* populations living in sympatry, and 3) between two additional species (*B. bifarius* and *B. vancouverensis*) living mainly in allopatry. We find that IoDs, defined by elevated *F*_ST_, recur in the same genomic locations associated with low recombination and centromeres in all three independent comparisons. Strikingly however, we find that the genomic landscape of divergence in the sympatric comparison displays distinct features that indicate that it has been shaped by differential gene flow. Firstly, a measure of absolute divergence, *d*_XY_, is elevated in IoDs in the sympatric but not the allopatric comparison. The *d*_XY_ statistic has been shown by simulations to be elevated in IoDs under conditions of differential gene flow (Cruickshank and Hahn 2014). Secondly, there is a more extensive drop in genetic variation in IoDs (∼2 Mb on an average) in both species of the sympatric pair compared with the allopatric pair. This may indicate a greater effect of selection in removing introgressed alleles at barrier loci in these regions. Thirdly, there is a markedly bimodal distribution of window-based *F*_ST_ in the sympatric pair, which is not present in the other comparisons, also reflecting the presence of extensive IoDs in this comparison. A similar distribution of *F*_ST_ has also been observed among *Heliconius* species that have undergone speciation without geographical isolation (Martin et al. 2013; Seehausen et al. 2014).

Another process that can result in IoDs with elevated *d*_XY_ is balancing selection in the ancestral population (Guerrero and Hahn 2017; Han et al. 2017). Although we cannot formally exclude the possibility that IoDs in the sympatric comparison represent ancient balanced polymorphisms, several observations strongly favor the differential gene flow model. Firstly, ancient balanced polymorphisms are not expected to be strongly associated with centromeres and regions of low recombination, which is observed for IoDs in all comparisons. Balanced polymorphisms are expected to be associated with loci involved in adaptation, which should not be strongly biased in their genomic locations. Secondly, we would not expect IoDs in the same genomic locations to represent ancient balanced polymorphisms in the sympatric comparison but not the allopatric one, considering that ancient balanced polymorphisms would be able to sort regardless of the presence of gene flow. Elevated *d*_XY_ in IoDs in the sympatric comparison therefore most likely indicates that they have acted as barriers to introgression during periods of gene flow.

The IoDs identified in the allopatric comparison appear most consistent with formation by linked selection in the absence of gene flow. No evidence for recent gene flow in the allopatric comparison is revealed by our data, as also found by (Ghisbain et al. 2020), although historical gene flow or low levels of ongoing gene flow where the ranges of the species overlap cannot be ruled out. The mechanism by which IoDs formed in the sympatric comparison is less clear. One possibility is that they were also formed by linked selection during periods when the species were isolated from each other. Another possibility is provided by the “continents” model of divergence-with-gene-flow, whereby selection against introgression at a large number of barrier loci across the genome mediated by the recombination landscape in the face of gene flow leads to IoDs (or continents) in regions of low recombination (Michel et al. 2010). More detailed modeling could potentially determine which of these scenarios is more feasible.

Our divergence time estimate of ∼396,000 years between *B. sylvicola* and *B. incognitus* coincides with a period of global cooling that was followed by rapid global warming around 340,000 years ago (Vimeux et al. 2002; Uemura et al. 2018). This could have been a driver of subpopulation isolation as cold-adapted alpine species likely became more isolated in mountain top habitats under warming (Hewitt 2000; Hines 2008). A subsequent period of cooling would then allow for secondary contact. It is therefore possible that a period of partial or complete geographic isolation facilitated the build-up of genetic incompatibilities by linked selection at IoDs which were then accentuated due to gene flow elsewhere in the genome during secondary contact. Quaternary climate oscillations are likely responsible for divergence in reproductive traits between populations of the red-tailed bumblebee (*B. lapidarius*) in Europe (Lecocq et al. 2013). Local fragmentation followed by gene flow during secondary contact could potentially be a common mode of speciation in high-altitude bumblebees, giving rise to cryptic species. Further modeling-based studies and empirical studies of more species are required to determine the validity and generality of this scenario. Knowledge of the distribution, ecology, and population history of *B. incognitus* is currently completely lacking. However, although details of the speciation process are unclear, our evidence suggests that the evolution of barrier loci in extended regions of low recombination near centromeres has promoted reproductive isolation between these two species.

### Recombination Mediates the Accumulation of Barriers to Gene Flow

Our results are compatible with other studies demonstrating that hybridizing natural populations harbor numerous genetic incompatibilities throughout their genomes (Schumer et al. 2014). Reduced introgression in regions of low recombination has been observed in hybrids of swordtail fish (Schumer et al. 2018). Similarly, a correlation between recombination rate and introgression has also been inferred in *Heliconius* butterflies (Martin et al. 2019), *Mimulus* monkey-flowers (Brandvain et al. 2014), house mice (Janoušek et al. 2015), and between Humans and Neanderthals (Juric et al. 2016). These observations could be due to the interaction of linkage and selection against introgression of genetic incompatibilities (Coughlan and Matute 2020). Selection against genetic incompatibilities in regions of low recombination is expected to remove introgressed alleles in a larger portion of the genome due to linkage (Schumer et al. 2018). This mechanism could also promote differentiation in regions of low recombination under conditions of gene flow. Regions of low recombination could also accumulate fixed genetic incompatibilities in the absence of gene flow due to the effects of linked selection, which could also lead to reduced introgression in these regions upon secondary contact (Ravinet et al. 2017).

Bumblebees may be a particularly good model systems to uncover the influence of genome architecture on species divergence due to their extremely high rates of recombination. The average recombination rate in the bumblebee *B. terrestris* has been estimated as ∼9 cM/Mb (Kawakami et al. 2019). High recombination rates have been estimated in other social insects, and appear to correlate with the degree of sociality (Wilfert et al. 2007) (rates in the highly social honeybee have been estimated as >20 cM/Mb; Kawakami et al. 2019). Importantly, in both honeybees and bumblebees, there is also an extreme reduction in recombination rates in centromeres (Kawakami et al. 2019) and we observe a clear association between regions of extremely low recombination near to centromeres and high divergence in the data we present here. The factors that determine variability along chromosomes appear to be constant among distantly related bee species (Jones et al. 2019). We therefore do not expect the landscape of recombination to be variable among the bumblebee species under investigation here, and expect the average rate to be similar to *B. terrestris*.

In addition to having low recombination rates, which enhances the effects of linked selection, IoDs in pericentromeric regions, as we observe here, could also be enhanced by genetic hitchhiking connected to the process of centromere drive (Crespi and Nosil 2013). This process results in rapid turnover of centromere satellite repeat sequences and proteins involved in the binding of centromeres to the spindle fibers during meiosis (Henikoff et al. 2001). Known speciation genes in Drosophila, OdsH (Bayes and Malik 2009) and Zhr (Sawamura et al. 1993), are both involved in interactions with satellite repeats. However, the IoDs are also gene rich, and therefore contain many functional sites that could be the focus of genetic hitchhiking or background selection, contributing to their elevated divergence.

Many studies of speciation genomics are focused on identifying specific genes that drive reproductive isolation by scanning the genome for IoDs (Ravinet et al. 2017). However, the interaction between linked selection and recombination rate variation can explain the presence of IoDs in genomic comparisons. This has led some authors to distinguish between “incidental” IoDs formed by linked selection in regions of low recombination but irrelevant for the speciation process and true IoDs that harbor loci involved in reproductive isolation that are resistant to gene flow (Poelstra et al. 2014; Vijay et al. 2016). However, in this study, we uncover evidence that IoDs that are found in regions of low recombination and appear to be resistant to gene flow in sympatry, indicating that the genome architecture is important in the formation of barriers to gene flow. The pervasiveness of this mechanism in nature is unclear. It is possible that a narrow set of conditions is required to generate IoDs with elevated *d*_XY_ indicating that there is often low statistical power to identify IoDs with elevated *d*_XY_, particularly for species with short divergence times (Cruickshank and Hahn 2014). It is therefore possible that differential gene flow between the genomes of young species is more common than expected based on analysis of IoDs in pairwise genome comparisons. As there is no evidence that gene flow is ongoing between these species it was not possible to directly measure its effects across the genome. However, this study is consistent with a growing number of others indicating that selection against gene flow between incipient species can be highly polygenic, and strongly influenced by genome architecture (Michel et al. 2010; Coughlan and Matute 2020). It also supports a multitude of recent genome-wide studies that attest to the pervasiveness of gene flow and permeability of species barriers in nature.

## Conclusion

We compared variation across the genomes of two recently diverged cryptic bumblebee species living in sympatry. This comparison revealed the presence of restricted genomic islands (IoD) with elevated levels of absolute divergence (*d*_XY_). This pattern suggests that the two species diverged under conditions of gene flow, which was restricted in regions of low recombination close to centromeres. These results imply that recombination rate variation could often be a crucial factor in determining the location of genomic barriers to gene flow between incipient species. We speculate that climatic fluctuations could be an important driver of speciation by this process in bumblebees with high-altitude habitats, whereby periods of warming lead to periodic population fragmentation at higher altitudes followed by secondary contact and differentiation under gene flow.

## Materials and Methods

### Genome Sequencing and Assembly

We generated a reference genome for *B. sylvicola* using ONT sequencing. DNA was extracted from a single male bee sampled from Niwot Ridge, CO using a salt-isopropanol extraction followed by magnetic bead purification to remove fragments <1,000 bp and to concentrate the sample for library preparation. Sequencing was performed on a MinION with two R9.4 flowcells using the RAD004 kit (ONT) starting with 3–400 ng DNA per run, resulting in a yield of 9.4 Gb with a total 2.5 million reads and a mean read length of 3.7 kb. We used a multistep approach to assemble the sequencing reads: downpore (https://github.com/jteutenberg/downpore, last accessed March 24, 2021) was used for adaptor trimming and splitting chimeric reads, trimmed reads were assembled using wtdbg2 using default settings (Ruan and Li 2020), then two rounds of the standalone consensus module Racon (https://github.com/isovic/racon, last accessed March 24, 2021) followed by further contig improvements with medaka v.0.4 (https://github.com/nanoporetech/medaka, last accessed March 24, 2021). For the medaka step, contigs of <20 kb were removed in order for the process to complete. The final polishing step involved two rounds of Pilon polishing (https://github.com/broadinstitute/pilon, last accessedMarch 24, 2021), whereby Illumina short reads were mapped to the assembly in order to correct the contigs around indels.

Long-range information from short-read sequencing of linked reads was obtained using 10× Genomics chromium technology. Sequencing was performed on the. A 10× GEM library was constructed from high-molecular weight DNA from the same bee as for the ONP sequencing according to the manufacturer’s recommended protocols. The resulting library was quantitated by qPCR and sequenced on one lane of a HiSeq 2500 using a HiSeq Rapid SBS sequencing kit version 2 to produce 150-bp paired-end sequences. We mapped the resultant reads to the assembly using Longranger v.2.1.4 and then ran Tigmint v1.1.2 to identify and correct errors in the assembly. ARCS+LINKS was used to scaffold the assembled contigs. We identified contigs that contained mitochondrial genes, and were therefore likely fragments of the mitochondrial genome, by running a BLAST search of *B. impatiens* mitochondrial genes across the assembly using BLAST+ v2.9.0. Any contigs containing two or more mitochondrial genes located within the expected distance of each other based on their locations on the mitochondrial genome were removed from the assembly, so that the final assembly did not contain partially assembled mitochondrial genome sequence. All contigs shorter than 10 kb were also removed from the assembly. We ran BUSCO v3.0.2b (Simão et al. 2015) on the assembly in order to assess its completeness using the hymenoptera_odb9 lineage set and species *B. impatiens*. We performed whole-genome synteny alignments between the *B. terrestris* chromosome-level genome assembly and our *B. sylvicola* contigs using Satsuma v.3 (Grabherr et al. 2010) to arrange *B. sylvicola* contigs into pseudochromosomes, with the assumption of high structural conservation between the species. We performed both de novo and guided transcriptome assemblies using reads from four different tissues: the abdomen, the head, the legs, and the thorax. Full details of the annotation pipeline can be found in supplementary methods, Supplementary Material online.

### Genome Features

All genome features were calculated over 20-kb nonoverlapping windows for each contig. Genome GC content was measured using a custom perl script. We used GenMap (https://github.com/cpockrandt/genmap, last accessed March 24, 2021) to calculate mappability (uniqueness of k-mers) for each position in the genome, using a k-mer size (k) of 150 and a mismatch tolerance (e) of 2, and then averaged the output across windows using a custom perl script. RepeatMasker output from the genome annotation step was also summarized over windows, giving the proportion of each window that was characterized as repeat sequence, using a custom perl script.

In order to identify putative centromeric repeats in the *B. sylvicola* genome, we ran centromere_seeker (https://github.com/cryancampbell/centromere_seeker, last accessed March 24, 2021) on the raw ONT reads. This pipeline runs tandem repeats finder (TRF) (Benson 1999) to identify the longest and most prevalent tandem repeat arrays in the sequences, which are likely centromeric repeats. We used BlastN (Altschul et al. 1990) to locate trimers of the identified 15-bp satellite in the genome to identify the likely locations of centromeres.

### Population Sampling

During the summer (July) of 2017 female worker bees from several species of *Pyrobombus* bumblebees were collected on seven mountains within the Rocky Mountains. Samples were collected with sweeping hand nets and kept in falcon tubes on cold packs in cool boxes for transport. Species identification was performed using a standard key (Williams et al. 2014). All samples were placed at −20 °C for approximately 10 min before being dissected. Thoraces were stored in 95% ethanol for DNA extraction. Sampling effort achieved 217 *B. sylvicola*, 67 *B. incognitus*, and 17 *B. bifarius* individuals. Our sampling was supplemented with sequencing data for other *Pyrobombus* bees from published data sets available on the NCBI sequence read archive. These included 21 samples of *B. melanopygus* from western USA (Tian et al. 2019) (NCBI accession no. PRJNA526235), four extra samples of *B. bifarius* also collected in the Rocky Mountains, and 17 samples of *B. vancouverensis* from north-western USA (NCBI accession no. PRJNA592825).

### Phenotypic Variation among Species

Measurements of intertegular distance (a proxy for body size) were made for all *B. sylvicola* and *B. incognitus* samples, as well as the 17 *B. bifarius* samples newly collected in Colorado as part of this study (supplementary table S1, Supplementary Material online). Intertegular distance was measured from scaled photographs of individual bees using ImageJ (https://imagej.nih.gov/ij/, last accessed 24 March 2021). The prementum and glossa were dissected from the head, mounted, and photographed for quantification using ImageJ. A subset of 69 samples defined as either *B. sylvicola* (*N* = 39) and *B. incognitus* (*N* = 30) by genetic clustering were randomly selected from across all sampling locations and examined morphologically in more detail. We characterized the shape of the malar space; the pile color between the antenna and above the ocelli; the size, color, and location of the ocelli relative to the supraorbital line; and the presence of black pile on the scutellum. We also recorded the color of abdominal segments. Other body parts were sacrificed for genetic material; therefore, additional traits could not be characterized.

### Population Sequencing and Variant Calling

For all samples, DNA was extracted from the thorax of worker bees using the Qiagen Blood and Tissue kit. Paired-end sequencing libraries were prepared with Nextera Flex and samples were sequenced on an Illumina HiSeq X. Illumina paired-end reads were mapped to our *B. sylvicola* genome assembly using the *mem* algorithm in BWA (Li and Durbin 2009). Mappings were piped to samtools (Li et al. 2009), where they were sorted by coordinate, written to bam files and indexed. Duplicate reads were marked and read groups were added in the bam files using the Picard suite of tools (https://broadinstitute.github.io/picard/, last accessed March 24, 2021). We used GATK to call variants, following their recommended Best Practices (https://gatk.broadinstitute.org/hc/en-us, last accessed March 24, 2021). Briefly, we ran HaplotypeCaller on each sample’s bam file to create an individual-specific gVCF file. All gVCFs were then processed by GenomicsDBImport on a per contig basis, followed by GenotypeGVCFs to call variants. Variants were filtered using a hard set of filters using the VariantFiltration tool with thresholds recommended in the GATK best practices. These filters assess quality scores, depth of coverage, strand bias, and position on reads of variants to only retain high-quality, high-confidence SNPs. The resultant filtered vcf files were filtered for biallelic SNPs only. As all but one of the *B. vancouverensis* samples were haploid males, we used these samples to filter out SNPs that were called heterozygous in haploids and therefore are errors likely due to mapping issues at these sites.

### Phylogenetic Analysis of PEPCK and COI Loci

In an attempt to identify the unknown species in our data set, we extracted sequences of the PEPCK gene from our WGS data sets to generate per-sample sequences. We created a VCF file from all gVCFs containing only variants found across the PEPCK gene (located on contig_001: 5,658,366–5,659,292 bp) and filtered these variants with the same set of filters detailed above. We then ran the GATK “SelectVariants” tool to create a separate VCF per sample for the PEPCK gene, containing all positions where each sample differed from the reference. We then ran the GATK “FastaAlternateReferenceMaker” tool on each of these VCFs to generate a sequence representing the PEPCK gene per sample based on the variants in each VCF. Manual inspection of mapping files was also performed to ensure sequences accurately reflected the evidence in the bam files. All sequences were concatenated into a single fasta file (see Supplementary Material online), along with published PEPCK sequences for seven bumblebee species from Martinet et al. (2019) and we generated a neighbor-joining tree from the sequences in SplitsTree4 v. 4.14.6. One thousand bootstrap replicates were performed to assess support.

As the mitochrondrial genome was incompletely assembled in our genome assembly, we generated sequences from the COI locus using PCR and Sanger sequencing. We used the following primers adapted from (Hebert et al. 2004): LepF1, 5′-ATTCAACCAATCATAAAGATATTGG-3′ and LepR1 5′-TAAACTTCTGGATGTCCAAAAAATCA-3′ for both PCR and sequencing. Thermal cycling conditions were as follows: one cycle of 1 min at 94 °C, six cycles of 1 min at 94 °C, 1 min and 30 s at 45 °C, and 1 min and 15 s at 72 °C, followed by 36 cycles of 1 min at 94 °C, 1 min and 30 s at 51 °C, and 1 min and 15 s at 72 °C, with a final step of 5 min at 72 °C. We processed sequences using CodonCode Aligner v. 9.0.1.3 and constructed phylogenetic trees in the same way as for the PEPCK locus.

### Recombination Rate Variation

We measured patterns of linkage disequilibrium in our *B. sylvicola* population genomic data set to infer the population-scale recombination rate, ρ, implemented in the program LDHat. A custom likelihood lookup table was created using the “complete” program. The “interval” program was used to estimate mean ρ across regions, running for 1.1 million iterations with chain sampling every 10,000 iterations, a burn-in of 100,000 iterations, and a block penalty of 1. Output from “interval” was summarized using “stat” and then converted into 20-kb nonoverlapping window averages across contigs using a custom perl script.

### Genetic Variation among Species

We carried out a PCA on a thinned set of likely independent SNPs. We retained one SNP every 10 kb and a minimum minor allele frequency of 0.01 using vcftools v. 0.1.15. The thinned vcf file was converted to a genlight object in the adegenet package in R v.4.0.2, via an intermediate plink raw format file. A PCA was carried out on the genlight object using the *glPca* function of adegenet. A neighbor-joining tree was generated for the same set of SNPs in SplitsTree4 v. 4.14.6. We also generated a neighbor-net network (Bryant and Moulton 2004), implemented in SplitsTree4 v. 4.14.6, based on the same genome-wide SNP set in order to check whether the evolutionary history of *B. sylvicola* and *B. incognitus* appears tree-like and whether it presented any evidence for hybridization.

### Diversity and Divergence

We calculated nucleotide diversity (*π*) within populations/species and relative (*F*_ST_) and absolute (*d*_XY_) divergence between populations/species in 20-kb nonoverlapping windows across the genome. We used scripts provided by Simon Martin (https://github.com/simonhmartin/genomics_general, last accessed March 24, 2021) for these analyses as they take into account the mixed ploidy among our samples. Per-window *F*_ST_ values were then standardized to a Z score (ZF_ST_) in order to be able to compare genomic landscapes of divergence among pairs with different divergence times, using the following formula:

$${\mathrm{ZF}}_{\mathrm{ST}}=\;\frac{W{\mathrm{indow}\;F}_{\mathrm{ST}}-\mathrm{Median}\;F_{\mathrm{ST}}\;}{\mathrm{SD}\;\mathrm{of}\;F_{\mathrm{ST}}}.$$

To measure branch-specific changes in allele frequency, we calculated the PBS (Yi et al. 2010) for each species. For this, we first obtained window-based *F*_ST_ measures for each species compared with an outgroup, *B. melanopygus*. All *F*_ST_ values for each 20-kb window were then transformed to estimates of divergence times, *T*, using the equation (Nielsen et al. 1998):

$$T=\;-\ln(1-F_{\mathrm{ST}}).$$

The length of the branch leading to each species could then be calculated using the following formula:

$$\mathrm{PBS}=\;\frac{T_{S1S2}+\;T_{S1S0}-T_{S1S2}}{2}.$$

Here, *S1* and *S2* refer to the two species being compared, and *SO* is the outgroup *B. melanopygus*. The PBS value gives an estimate of the amount of divergence in terms of allele frequency change specific to a particular species (*S1*) since divergence from its common ancestor with the second species (*S2*).

### Divergence Time Estimates

Under a model of neutral divergence of two populations from a common ancestor, *F*_ST_ can be converted into an estimate of time since divergence, *T*, where *T *=* t*/3*N*_e_. Here, *t* is the number of generations since the two populations diverged and *N*_e_ is the effective size of each of the populations (Nielsen et al. 1998). Multiplying *N*_e_ by three is appropriate for haplodiploids. We estimated divergence time between *B. sylvicola* and *B. incognitus* and between *B. bifarius* and *B. vancouverensis* by calculating *T* from *F*_ST_ across all regions of the genome sitting outside of the identified IoDs and then took the mean *T*. We estimated effective population sizes using an estimate of the population mutation rate, Watterson’s estimator (*θ*_w_), using the following equation:

$$\theta_{w}=\frac{K}{a_{n}}.$$

Here, *K* is the number of segregating sites in the species and *a_n_* is the (*n* − 1)th harmonic number. Values of *θ*_w_ were then used to calculate *N*_e_ for each species using the equation 3 *N*_e_ = *θ*/*µ*, where *µ* is the mutation rate. We used a value of *µ* = 3.6 × 10^−9^, a direct estimate for *B. terrestris* (Liu et al. 2017). Multiplying *T* by 3*N*_e_ provided us with an estimate of the number of generations since the two species diverged (*t*). Assuming a generation time of 1 year, this estimate translates directly to the number of years since divergence. We calculated 95% confidence intervals around each estimate by bootstrapping the values of *T* from the 20-kb window estimates using 5,000 bootstrap replicates with the *boot* package in *R* v.4.0.2.

### Characterizing Islands of Divergence

We characterized 20-kb windows with ZF_ST_ values >2 (2 standard deviations above the median) as highly divergent separately for each species. Highly divergent windows within 60 kb of each other were then merged into single blocks. We classified divergent blocks greater than 100 kb in length as IoDs in each pair. For the within-species and sympatric comparisons, any two IoDs within 1 Mb of each other were merged into single IoDs as they likely are part of the same divergent region but small drops in ZF_ST_ in between meant they were not brought together in the previous step. We then defined all 20-kb windows as either “IoD” or “background” for each population/species comparison separately and compared window measures of *π*, *d*_XY_, PBS, recombination rate (ρ/kb), GC content, mappability, and repeat content inside and outside of IoDs for each pair using Wilcoxon rank sum tests in R v.4.0.2.

We used permutation tests implemented with the R package *regioneR* (Gel et al. 2016) to assess significance of overlap in the positions of IoDs between comparisons in a pairwise fashion. We used the “randomizeRegions” function with the “per.chromosome” option to randomize the location of each IoD along each pseudochromosome while maintaining its size. We performed 1,000 permutations and measured significance of the observed overlap by comparing it to a distribution of overlap in randomly positioned IoDs. Calculated *Z*-scores gave a measure of the strength of the association. We used this same method to assess whether exon content of IoDs is greater or less than that expected by chance, where the positions of exons and IoDs were randomized across the genome while maintaining their size and 1,000 permutations were used to assess significance of the observed overlap.

To assess how diversity and divergence change when moving away from the centers of IoDs, we took the positions of the center of each IoD and used custom perl scripts to calculate average *π*, ZF_ST_, and *d*_XY_ in 20-kb steps up to 5 Mb away from each center for the within-species, sympatric, and allopatric comparisons separately.

### Measuring Overlap between IoDs and Centromere Repeats

We measured overlap in the positions of IoDs and putative centromere repeat sequence for each comparison, using permutation tests in *regioneR* as described above. Here, the positions of both IoDs and repeats were randomized along each pseudochromosome while maintaining their size and 1,000 permutations were used to assess significance of the observed overlap. We used *Z*-scores to assess the effect size.

### Uncovering Evidence for Structural Variants between Species

We used the program manta (Chen et al. 2016) to look for evidence of structural variants (SVs) between *B. sylvicola* and *B. incognitus* genomes. Manta uses mapped paired-end sequencing reads to discover, assemble, and score large-scale SVs. In particular, we wanted to test whether there was evidence that the IoDs we identified could be the result of large inversions. As Manta is designed to run on a small set of samples, we ran it on the bam files of 12 *B. sylvicola* and 12 *B. incognitus* samples using default settings and then filtered the output for inversions that were fixed between the two species.

## Supplementary Material

Supplementary data are available at *Molecular Biology and Evolution* online.

## Supplementary Material

msab086_Supplementary_Data
