# Supplementary material for: Genetic Barriers to Historical Gene Flow between Cryptic Species of Alpine Bumblebees Revealed by Comparative Population Genomics
Source: Mol Biol Evol. 2021 Apr 6;38(8):3126–43. doi: 10.1093/molbev/msab086 (PMC8321533; doi:10.1093/molbev/msab086)
Supplement: msab086_Supplementary_Data [file msab086_supplementary_data.zip › bombus_IoD_supp_info_postrev.pdf]

SUPPLEMENTARY INFORMATION

Genetic barriers to historical gene flow between cryptic species of alpine bumblebees revealed by comparative population genomics

Matthew J. Christmas[1], Julia C. Jones[1,2], Anna Olsson[1], Ola Wallerman[1], Ignas Bunikis[3], Marcin Kierczak[4], Valentina Peona[5], Kaitlyn M. Whitley[6,7], Tuuli Larva[1], Alexander Suh[5,8], Nicole E. Miller-Struttmann[9], Jennifer C. Geib[6], Matthew T. Webster[1]

1) Department of Medical Biochemistry and Microbiology, Science for Life Laboratory, Uppsala University, Uppsala, Sweden
2) School of Biology and Environmental Science, University College Dublin, Dublin, Ireland
3) Department of Immunology, Genetics and Pathology, Science for Life Laboratory, Uppsala University, Uppsala, Sweden
4) Dept of Cell and Molecular Biology, National Bioinformatics Infrastructure Sweden, Science for Life Laboratory, Uppsala University, Uppsala, Sweden
5) Department of Organismal Biology – Systematic Biology, Uppsala University, Uppsala, Sweden
6) Department of Biology, Appalachian State University, Boone, North Carolina, USA
7) U.S. Department of Agriculture, Agriculture Research Service, Charleston, South Carolina, USA
8) School of Biological Sciences, University of East Anglia, Norwich Research Park, Norwich, UK
9) Biological Sciences Department, Webster University, St. Louis, Missouri, USA

Corresponding author: Matthew Webster, matthew.webster@imbim.uu.se

Supplementary Figures

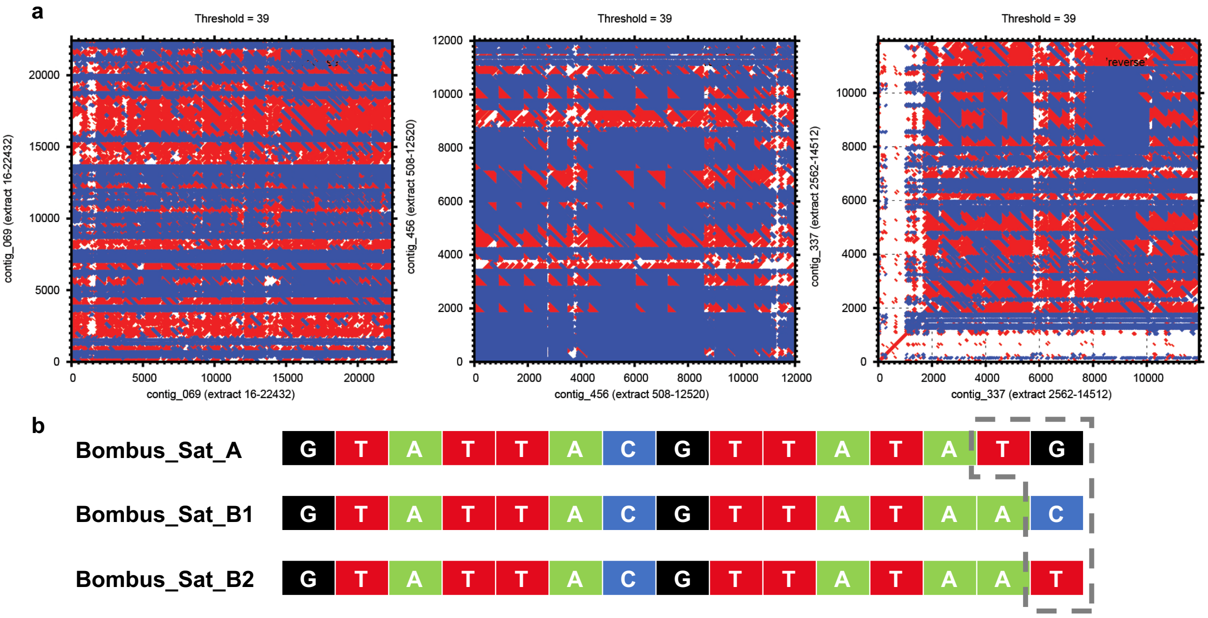

Supplementary figure S1: (a) Dot plots of self-alignments of the three longest tandem repeat arrays identified in the *Bombus sylvicola* genome. The repeats consist of (b) three 15 bp subunits which differ by one or two nucleotides (indicated by dashed grey line). Tandem repeat arrays of this sort commonly occur at contig boundaries, strongly associate with the locations of islands of divergence, and are strong candidates for centromere-associated repeats. In (a), red lines show forward alignments and blue lines show reverse alignments.

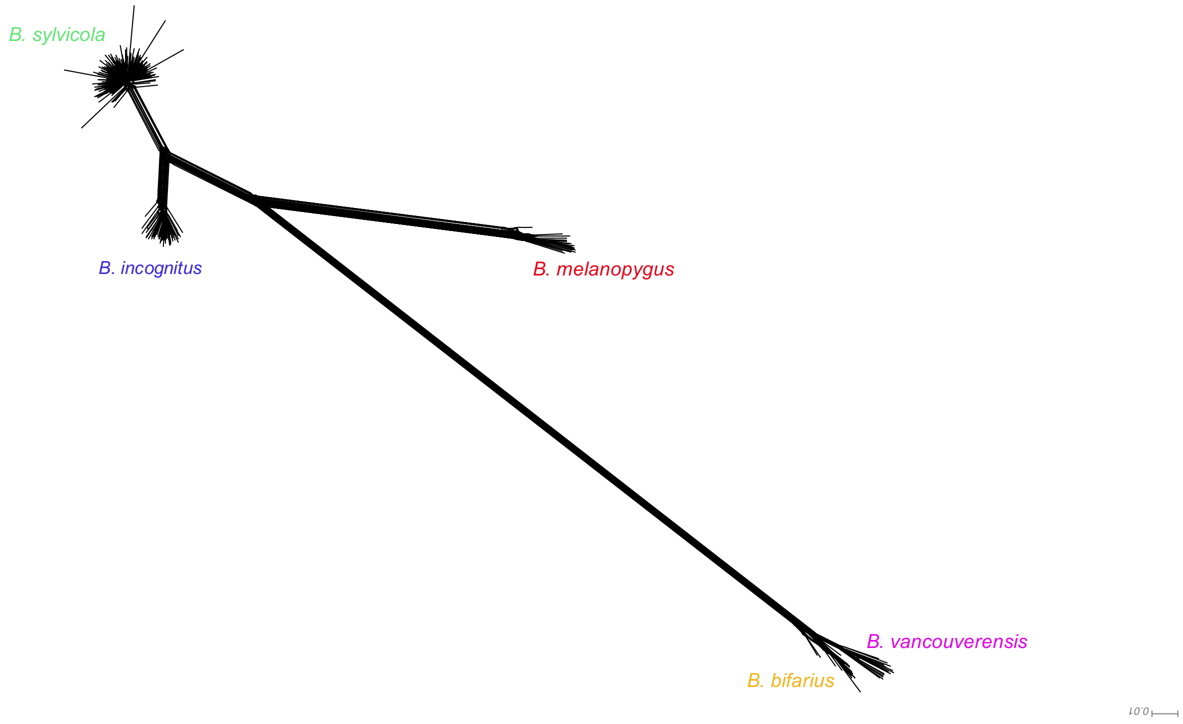

Supplementary figure S2: A neighbour-net network based on genome-wide SNPs thinned for one SNP every 10 kbp of the five *Pyrobombus* species included in this study reveals that the evolutionary history of *B. sylvicola* and *B. incognitus* is tree-like and does not present any evidence for hybridisation with another species.

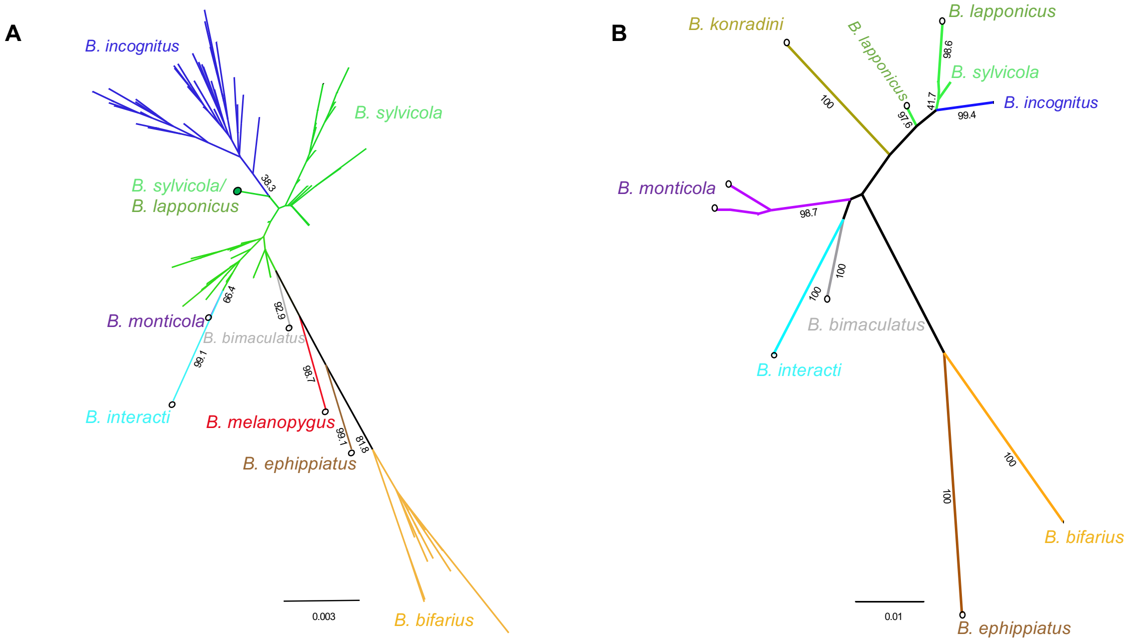

Supplementary figure S3: Neighbour-joining trees based on (A) 925 bp partial CDS of the PEPCK gene and (B) 449 bp partial sequence of the mitochondrial COI gene for a set of Pyrobombus species. Trees were generated in SplitsTree4 v.4.16.2. Branch support percentages are shown on branches leading to each species and were generated using bootstrapping with 1,000 replicates. Branches with open circles at the tips represent samples from a previous study (Martinet et al. 2019). The closed green circle in (A) indicates a tip representing five identical sequences, three from the current study identified as *B. sylvicola*, and two from Martinet et al. 2019 identified as *B. sylvicola* and *B. lapponicus.* The newly identified cryptic species described in the current study, *B. incognitus*, is represented by the dark blue branches. *Bombus interacti* (light blue) was recently discovered by Martinet et al. 2019, but this analysis strongly suggests that it is a different species to *B. incognitus*.

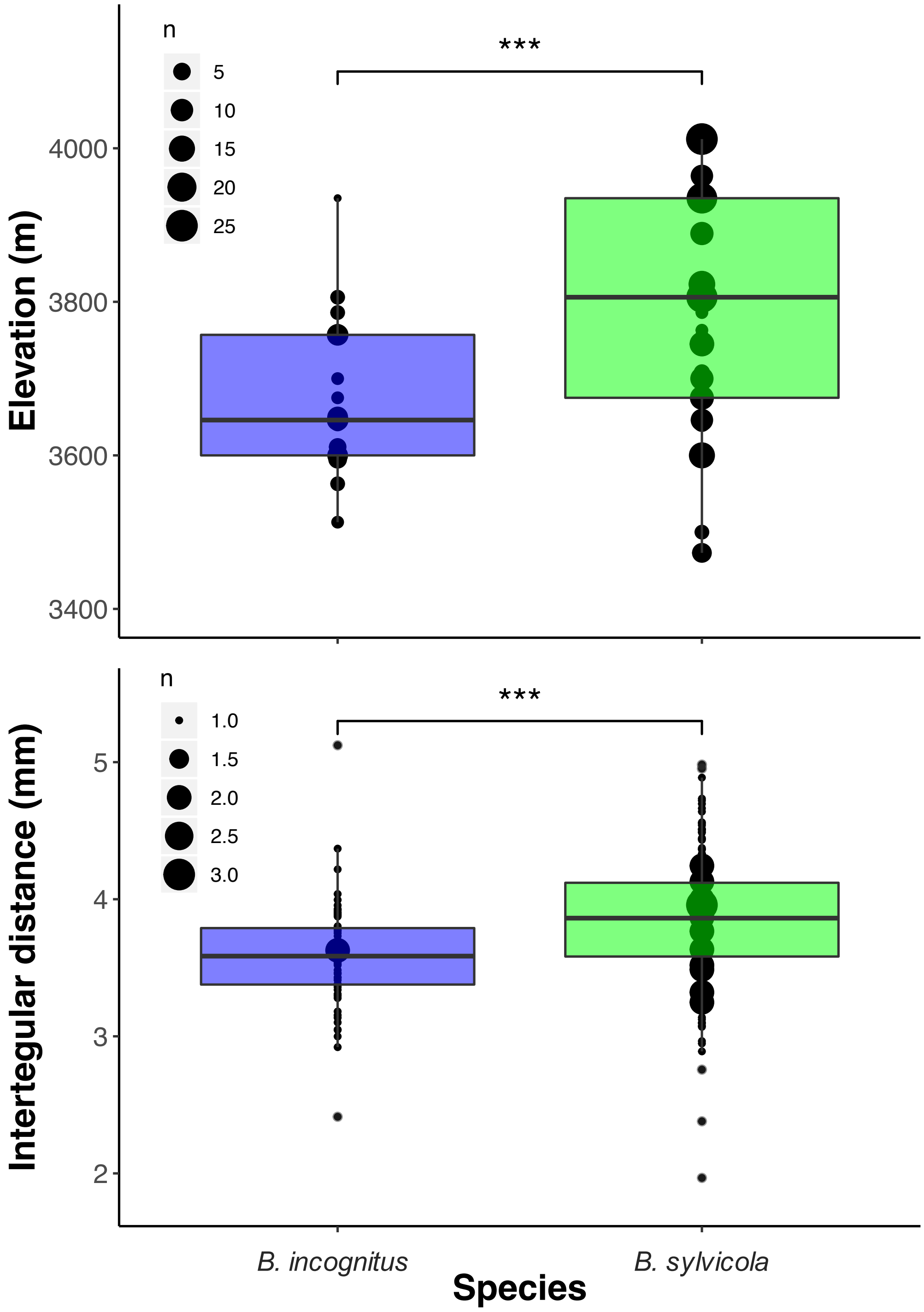

Supplementary figure S4: Boxplots comparing (a) differences in elevation samples were collected at and (b) intertegular distance, a proxy for body size, between *Bombus sylvicola* and *B. incognitus*. Boxes represent first quartile, median, and third quartile. The distributions were significantly different in both cases, as indicated by the asterisks (Wilcoxon rank sum tests, $p < 0.001$), although there is clear overlap in both size and elevation between the species.

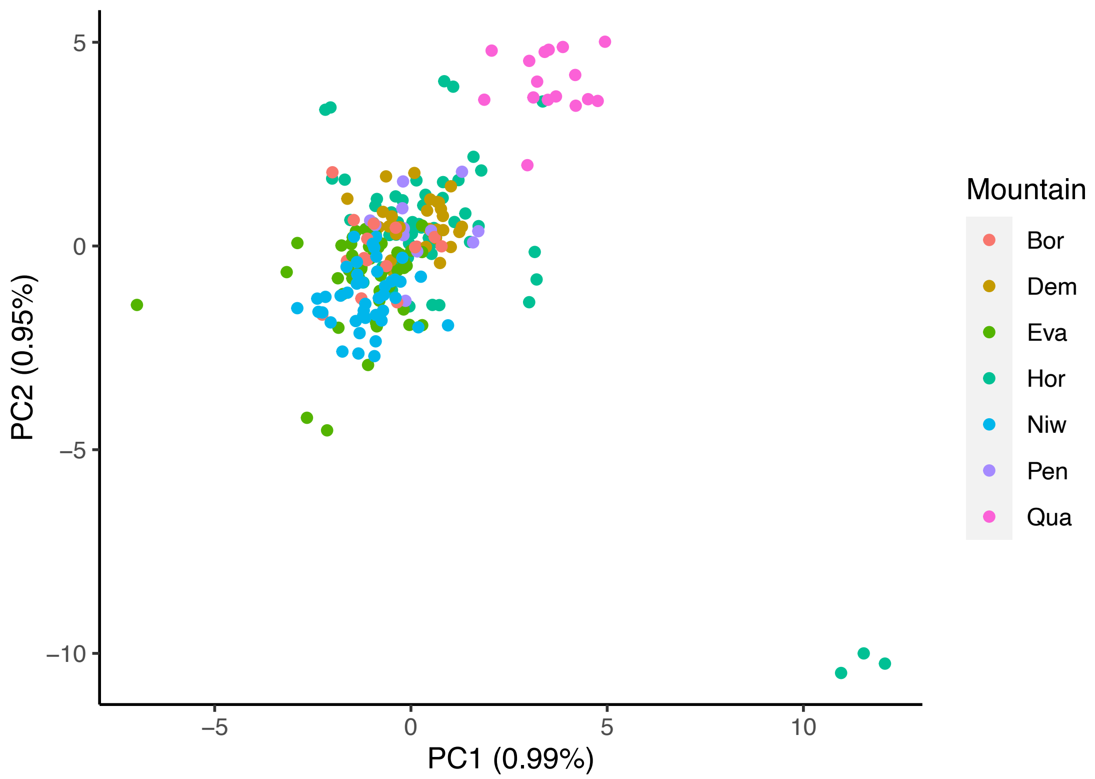

Supplementary figure S5: The first two principal components of a principal component analysis (PCA) of the 217 *Bombus sylvicola* samples included in this study, based on a genome-wide SNP set thinned for one SNP every 5,000 bases (total 49,339 SNPs). Percentages show the percentage of variance explained by each axis. Samples are coloured by the mountain they were collected on, where Bor = Boreas Mountain, Dem = Mount Democrat, Eva = Mount Evans, Hor = Horseshoe Mountain, Niw = Niwot Ridge, Pen = Pennsylvania Mountain, Qua = Quail Mountain. All are within the Rocky Mountains, CO, USA.

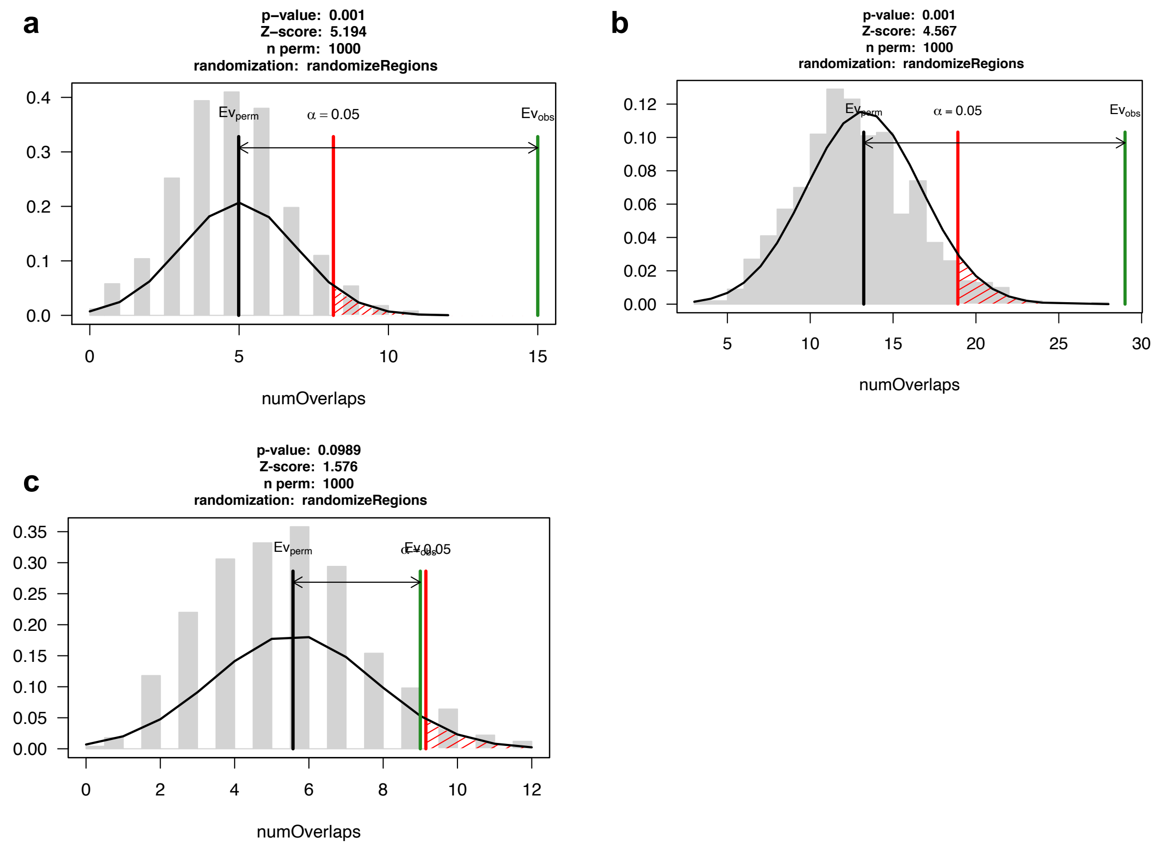

Supplementary figure S6. Significance of overlap in islands of divergence (IoDs) assessed using 1,000 randomised permutations for IoDs identified in a) *B. sylvicola* Niwot Ridge – Quail Mountain populations and *B. sylvicola – B. incognitus*, b) *B. sylvicola – B. incognitus* and *B. bifarius – B. vancouverensis*, and c) *B. sylvicola* Niwot Ridge – Quail Mountain populations and *B. bifarius – B. vancouverensis* Grey bars represent the distribution of overlaps from the 1,000 permutations, black vertical lines show mean number of overlaps for the 1,000 permutations, red vertical lines indicate significance threshold (p = 0.05), green lines indicate number of observed overlaps. Z-scores were computed as difference between observed overlaps and mean overlap of permutations divided by the standard deviation of the permutations.

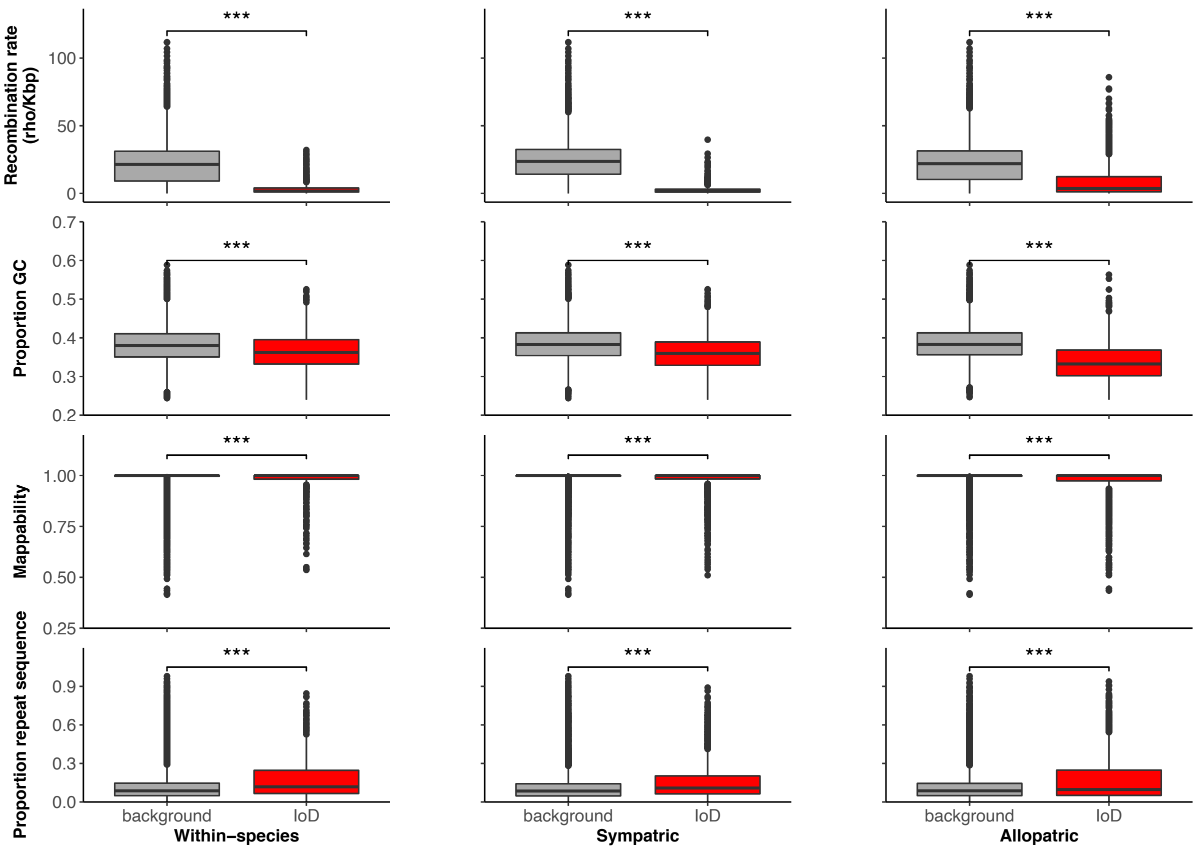

Supplementary figure S7: Boxplots showing differences in recombination rate, GC content, mappability, and repeat content inside and outside of islands of divergence (IoDs) for the within-species pair of *Bombus sylvicola* Niwot Ridge and Quail Mountain, the sympatric pair of *B. sylvicola* and *B. incognitus*, and the allopatric pair of *B. bifarius* and *B. vancouverensis*. In all cases, the distributions were significantly different, as indicated by the asterisks (Wilcoxon rank sum tests, $p < 0.001$). These results strongly suggest that regions of heightened relative divergence are generally located in low recombining regions of the genome.

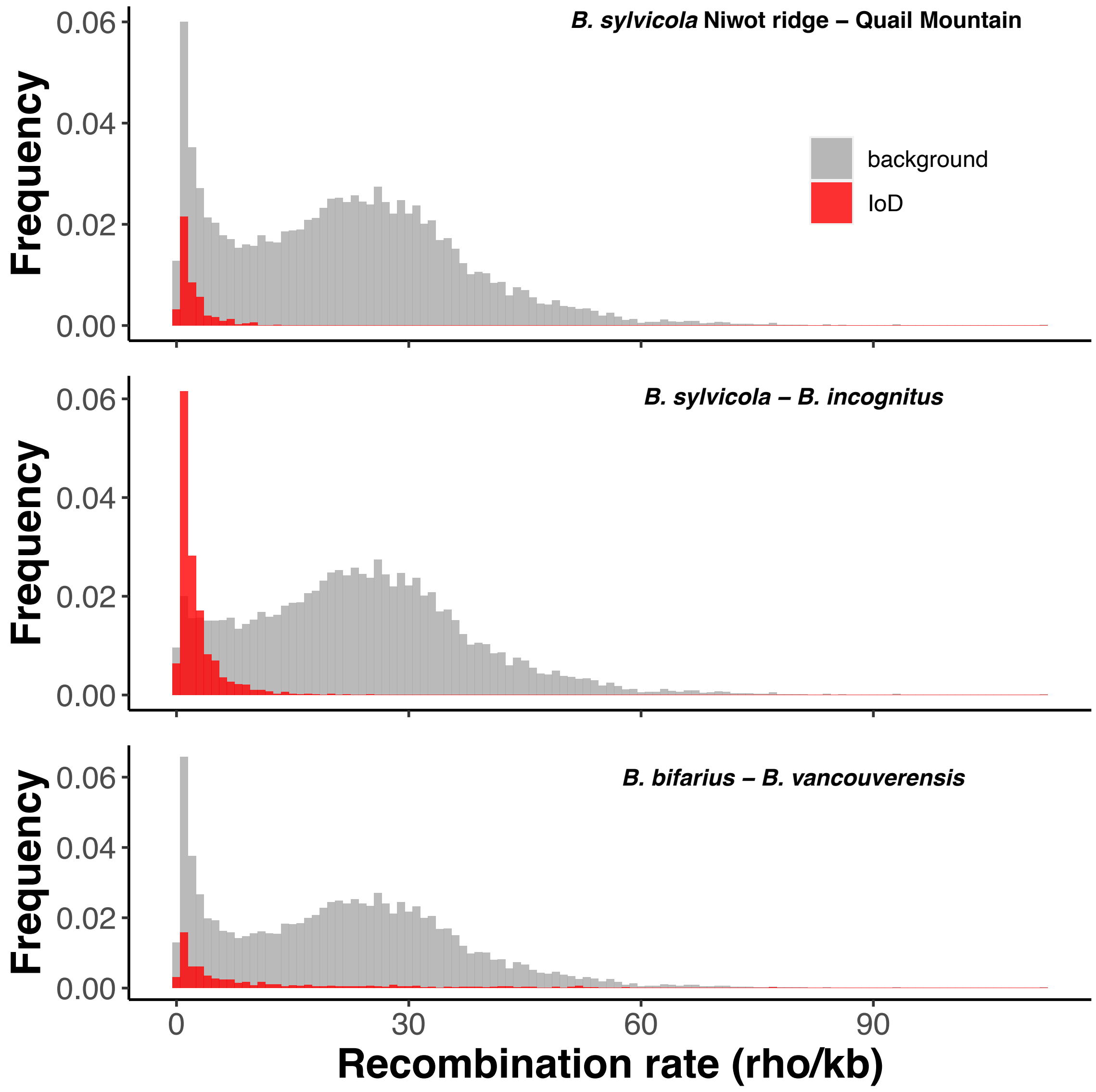

Supplementary figure S8: Histograms of recombination rate variation (rho/kb) across the genome, measured in 20 Kbp non-overlapping windows, inside (red) and outside (grey) of IoDs identified in (A) the within-species pair of *Bombus sylvicola* Niwot Ridge and Quail Mountain, (B) the sympatric pair of *B. sylvicola* and *B. incognitus*, and (C) the allopatric pair of *B. bifarius* and *B. vancouverensis*.

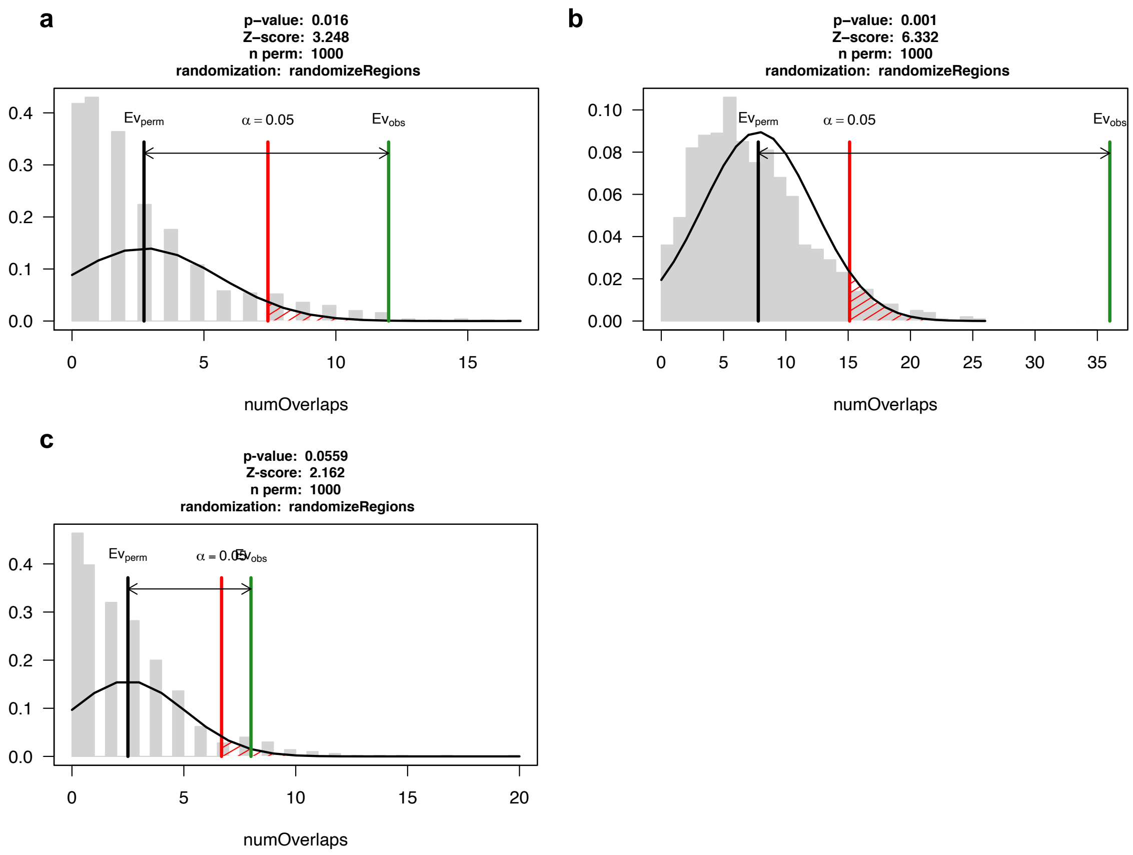

Supplementary figure S9. Significance of overlap in the locations of islands of divergence (IoDs) and putative centromeric repeats. Locations of IoDs and repeats were randomised with 1,000 permutations. a) *B. sylvicola* Niwot Ridge – Quail Mountain comparison, b) *B. sylvicola* – *B. incognitus* comparison, and c) *B. bifarius* – *B. vancouverensis* comparison. Grey bars represent the distribution of overlaps from the 1,000 permutations, black vertical lines show mean number of overlaps for the 1,000 permutations, red vertical lines indicate significance threshold (p = 0.05), green lines indicate number of observed overlaps. Z-scores were computed as difference between observed overlaps and mean overlap of permutations divided by the standard deviation of the permutations.

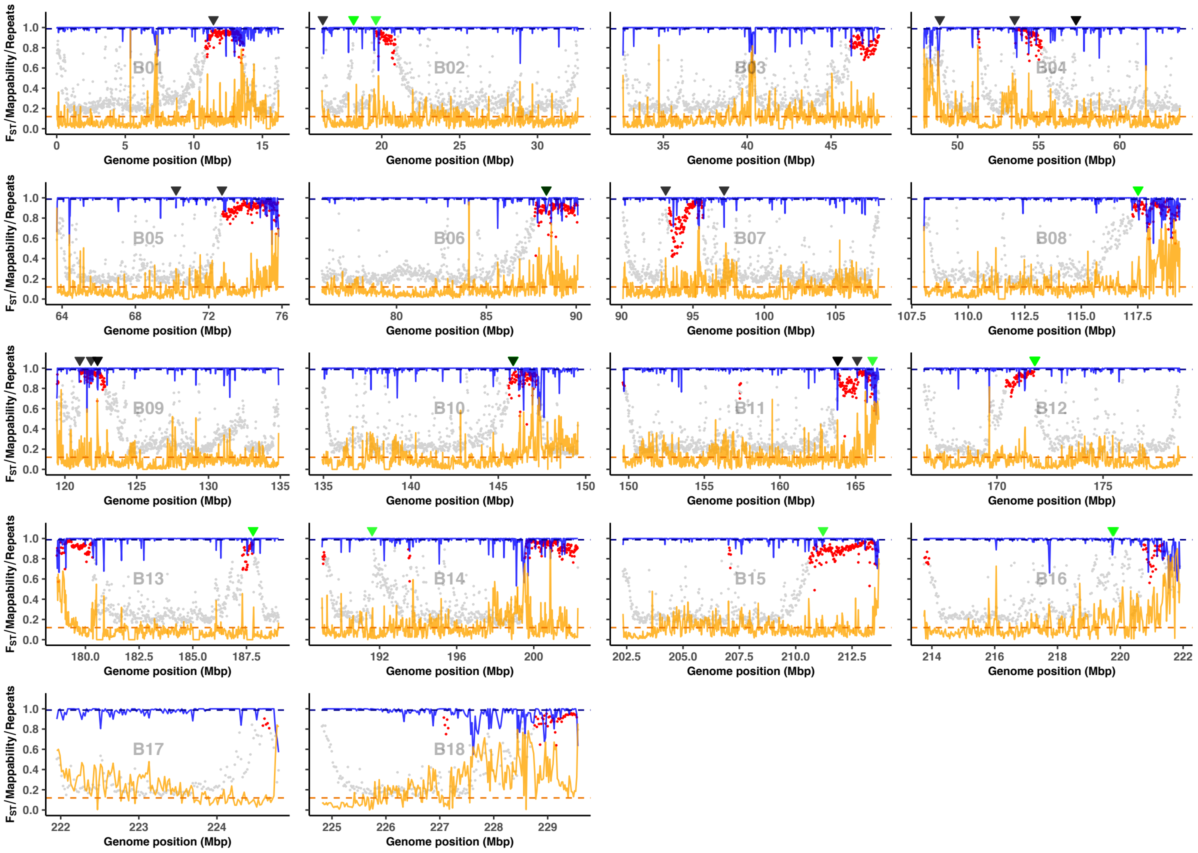

Supplementary figure S10: Per-pseudochromosome plots showing F_{ST} between *Bombus sylvicola* and *Bombus incognitus* measured in 20 Kbp non-overlapping windows (grey and red dots), mappability (blue lines) and repeat content (yellow lines). Red dots represent windows located in islands of divergence (ZF_{ST} > , longer than 100 Kbp). Black and green arrows represent locations of putative centromere tandem repeat arrays that are > 1 Kbp and < 1 Kbp respectively. Whilst repeat content tends to be higher and mappability tends to be lower inside IoDs compared to the rest of the genome (see main text), high divergence is still observed inside IoDs across regions where repeat content and mappability match that of the rest of the genome, strongly suggesting that what we observe is not an artefact of the repetitive nature of pericentromeric regions.

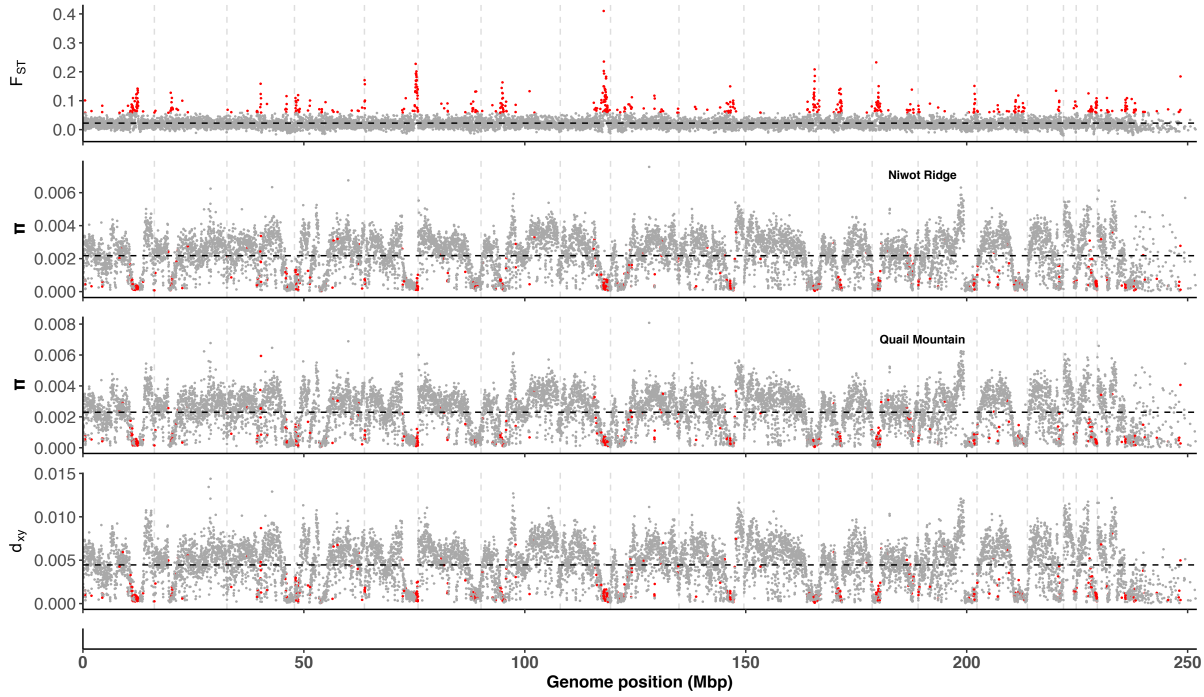

Supplementary figure S11: Genome-wide plots of diversity and divergence for the within-species comparison of *Bombus sylvicola* Niwot Ridge and Quail Mountain populations measured in 20 Kbp non-overlapping windows. (a) relative divergence (F_{ST}), (b) nucleotide diversity (π) for the Niwot Ridge population, (c) nucleotide diversity (π) for the Quail Mountain population, (d) absolute divergence (d_{XY}) between the populations. Red dots represent 20Kbp windows that are found within islands of divergence defined by $ZF_{ST} > 2$ and length > 100 Kbp.

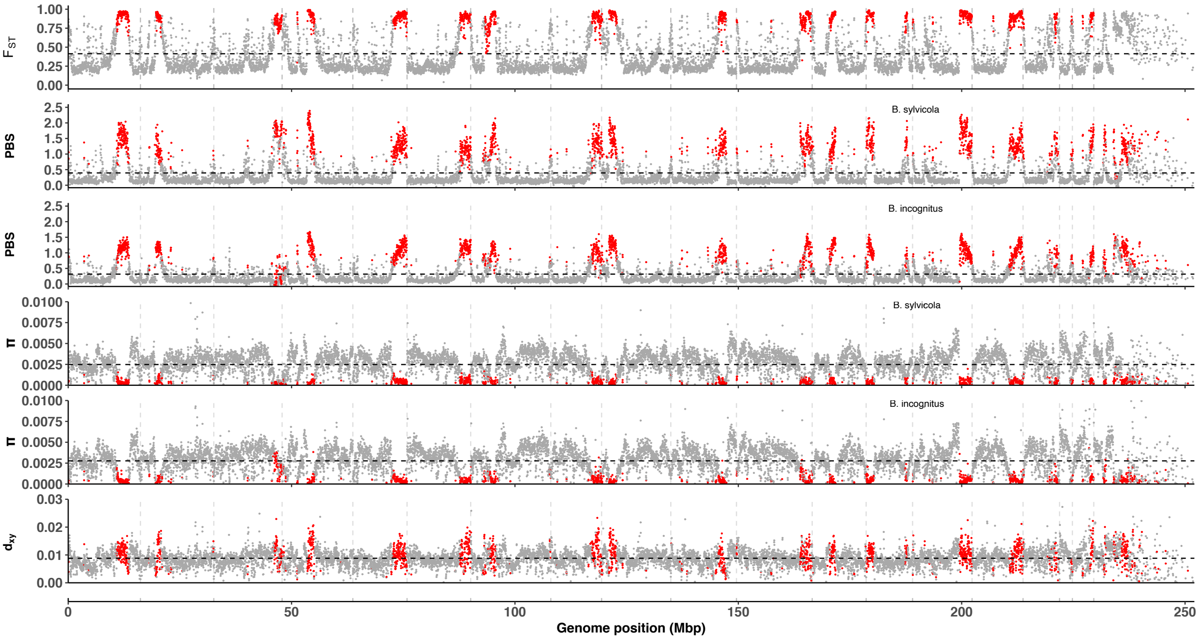

Supplementary figure S12: Genome-wide plots of diversity and divergence for the sympatric comparison of *Bombus sylvicola* and *B. incognitus* measured in 20 Kbp non-overlapping windows. (a) relative divergence (F_{ST}), (b) nucleotide diversity (π) for *B. sylvicola*, (c) nucleotide diversity (π) for *B. incognitus*, (d) absolute divergence (d_{XY}) between the species. Red dots represent 20Kbp windows that are found within islands of divergence defined by ZF_{ST} > 2 and length > 100 Kbp.

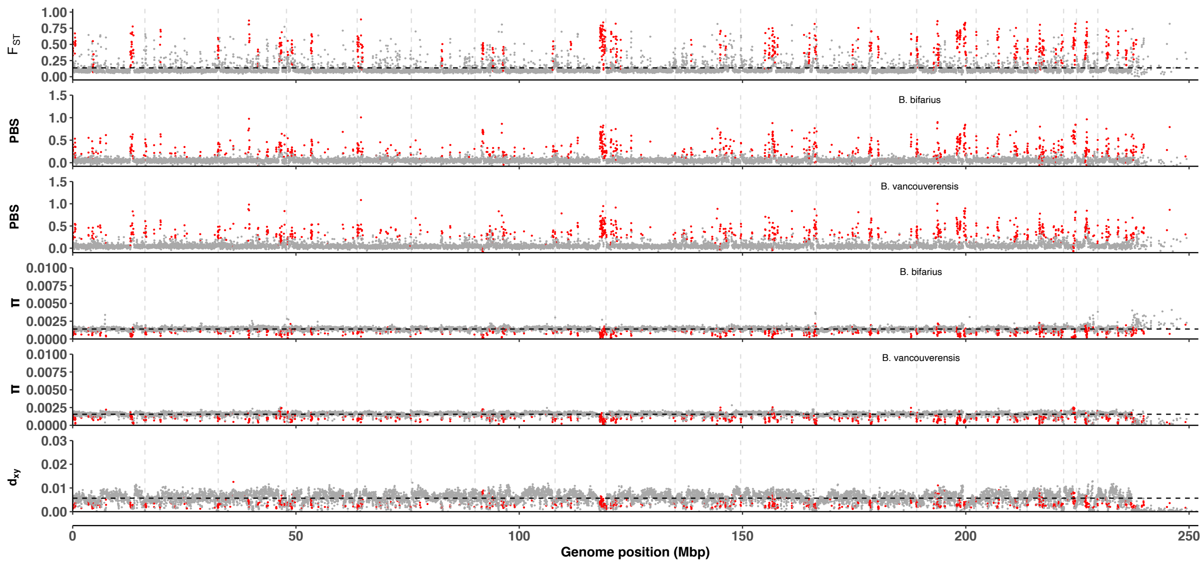

Supplementary figure S13: Genome-wide plots of diversity and divergence for the allopatric comparison of *Bombus bifarius* and *B. vancouverensis* measured in 20 Kbp non-overlapping windows. (a) relative divergence (F_{ST}), (b) nucleotide diversity (π) for *B. bifarius*, (c) nucleotide diversity (π) for *B. vancouverensis*, (d) absolute divergence (d_{XY}) between the species. Red dots represent 20Kbp windows that are found within islands of divergence defined by ZF_{ST} > 2 and length > 100 Kbp.

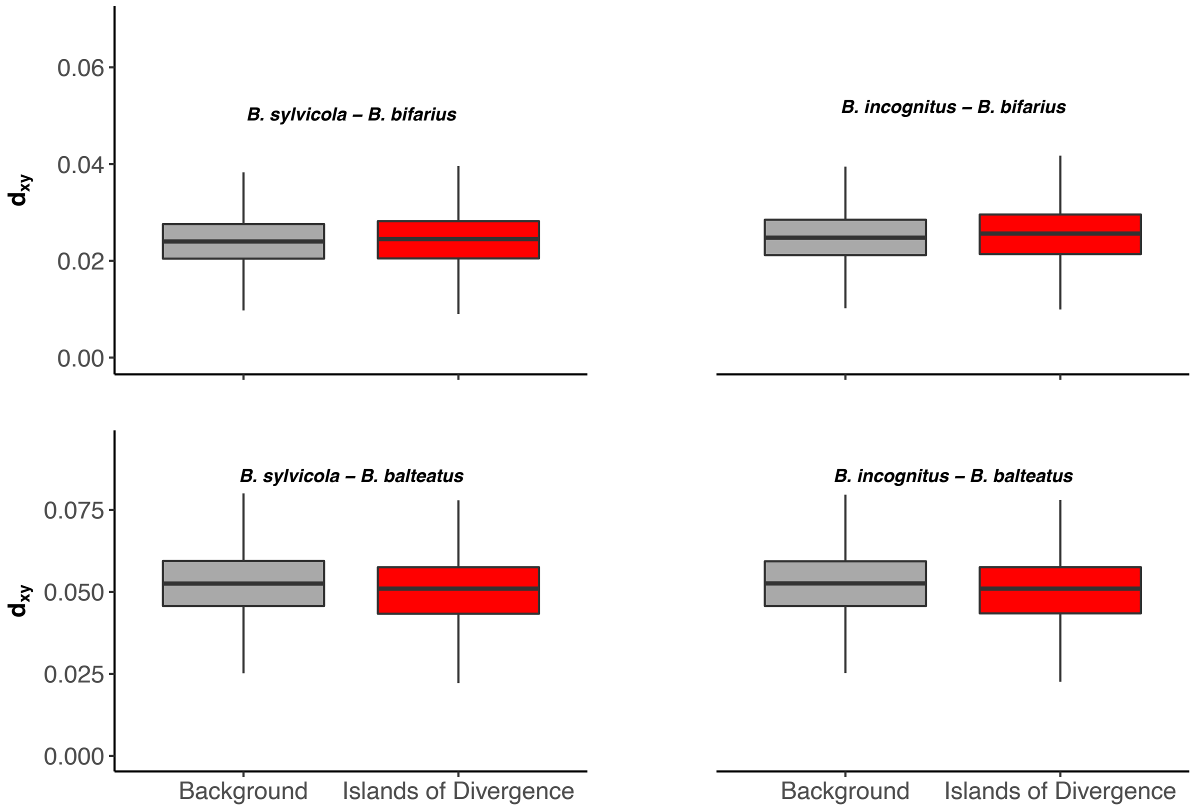

Supplementary figure S14: Boxplots comparing absolute divergence (d_{XY}) inside and outside of islands of relative divergence (IoDs, where $ZF_{ST} > 2$) identified in *B. sylvicola – B. incognitus* comparison for *B. sylvicola* and *B. incognitus* compared to *B. bifarius* and *B. balteatus*. None of the comparisons were significantly different at $p < 0.05$ (Wilcoxon rank sum test), suggesting that mutation rates within IoDs are not greater than those outside of IoDs and that elevated d_{XY} in IoDs between *B. sylvicola* and *B. incognitus* is due to differential gene flow.

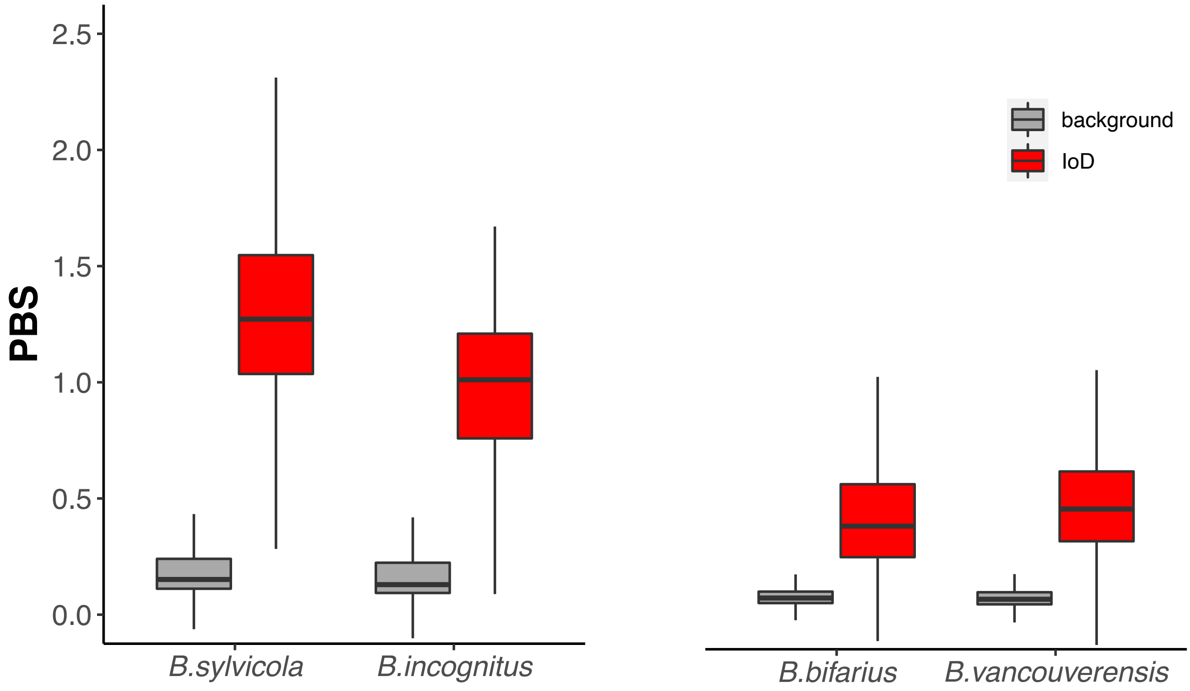

Supplementary figure S15: Differences in Population Branch Statistic (PBS) inside and outside of islands of relative divergence (IoDs, where $ZF_{ST} > 2$) for four *Pyrobombus* species. *Bombus sylvicola* and *B. incognitus* occur in sympatry whereas *B. bifarius* and *B. vancouverensis* occur in allopatry.

Supplementary Methods

Genome annotation pipeline

Overview

The NBIS (National Bioinformatics Infrastructure, SciLifeLab, Sweden) annotation pipeline consists of the following stages:

- collecting reference proteins and transcriptomes,
- modelling repeat sequences to mask the genome,
- preparing the first evidence-based gene build and generating annotation (evidence build),
- filtering out poor/incomplete gene models from the evidence build,
- training *ab-initio* gene finders using the filtered gene models,
- preparing the *ab-initio* and evidence-driven gene build (hybrid build),
- assessing quality of the different annotations,
- performing functional annotation of the hybrid build using Blast matches against Uniprot/Swissprot and results from *InterproScan*.

1) Pre-processing

Before starting the annotation process, we assessed the genome completeness using BUSCO (v3.0.2b). We used the *hymenoptera_odb10* as the reference lineage. We obtained the following results:

- C:97.9%[S:97.7%,D:0.2%],F:0.6%,M:1.5%,n:5991
- 5865 Complete BUSCOs (C)
- 5856 Complete and single-copy BUSCOs (S)
- 9 Complete and duplicated BUSCOs (D)
- 34 Fragmented BUSCOs (F)
- 92 Missing BUSCOs (M)
- 5991 Total BUSCO groups searched

2) Transcriptome assembly

We performed both *de novo* and guided transcriptome assemblies using reads coming from 4 different tissues: the abdomen, the head, the legs and the thorax. The transcriptome assembly pipeline consisted of the following steps:

· Read QC. FastQC was used to summarise reads properties and to check for common issues relating to sequence content and quality.

· Read trimming using fastp (v. 0.20.0). Reads were trimmed for adapter read-through and a QC summary is then provided.

· Guided assembly:

- Hisat2 Build: Builds an index database for the input genome. (Hisat2 v. 2.1.0; samtools v. 1.9)
- Hisat2: Align trimmed reads to the genome.

- Stringtie (v. 2.0): Assemble transcripts from the aligned reads.

· Generation of a summary report using MultiQC (v. 1.8).

In addition to the guided assemblies, we also performed a *de novo* assembly for reads coming from each of the 4 tissues. To this end, we used Trinity (v. 2.0.4) software.

3) Building repeats library

For each genome we created a species specific repeat library modelled using the RepeatModeler package (ver. DEV; -engine ncbi -pa 35). As repeats can be part of actual protein-coding genes, the candidate repeats modeled by RepeatModeler were vetted against our proteins set (minus transposons) to exclude any nucleotide motif stemming from low-complexity coding sequences. From the repeats library, identification of repeat sequences present in the genome was performed using RepeatMasker (open-4.0.9

4) Evidence build

As protein evidence, we used curated protein sequences that were collected from the Uniprot Swiss-Prot database (downloaded on 2019-11; 561 356 proteins).

Gene builds were computed using the MAKER package, version 3.01.02, which includes the following software : exonerate 2.0.4, blast 2.9.0, RepeatMasker 4.0.9_p2, BioPERL 1.7.2, Augustus 3.3.3, tRNAscan 1.4 and Snap (version 2006-07-28).

An evidence-guided build was computed by allowing the MAKER software to construct gene models directly from both aligned transcript sequences and reference proteins. Evidence builds generally closely reflect the information provided by the available sequence data and try to condense them to synthesize consensus transcript structures. However, this approach is vulnerable to missing or incomplete sequence material, as well as incorrect sequence in the transcriptome data.
